# *Aeromonas* species isolated from aquatic organisms, insects, chicken, and humans in India show similar antimicrobial resistance profiles

**DOI:** 10.3389/fmicb.2022.1008870

**Published:** 2022-12-01

**Authors:** Saurabh Dubey, Eirill Ager-Wick, Jitendra Kumar, Indrani Karunasagar, Iddya Karunasagar, Bo Peng, Øystein Evensen, Henning Sørum, Hetron M. Munang’andu

**Affiliations:** ^1^Section of Experimental Biomedicine, Department of Production Animal Clinical Sciences, Faculty of Veterinary Medicine, Norwegian University of Life Sciences, Ås, Norway; ^2^College of Fisheries, Acharya Narendra Deva University of Agriculture and Technology, Uttar Pradesh, India; ^3^Nitte University Centre for Science Education and Research, Mangaluru, India; ^4^State Key Laboratory of Biocontrol, Guangdong Key Laboratory of Pharmaceutical Functional Genes, School of Life Sciences, Southern Marine Science and Engineering Guangdong Laboratory (Zhuhai), Sun Yat-sen University, Higher Education Mega Center, Guangzhou, China; ^5^Department of Paraclinical Sciences, Faculty of Veterinary Medicine, Norwegian University of Life Sciences, Ås, Norway; ^6^Faculty of Biosciences and Aquaculture, Nord University, Bodø, Norway

**Keywords:** *Aeromonas*, resistance, plasmids, integrase, beta lactam, antimicrobials, transposase genes

## Abstract

*Aeromonas* species are Gram-negative bacteria that infect various living organisms and are ubiquitously found in different aquatic environments. In this study, we used whole genome sequencing (WGS) to identify and compare the antimicrobial resistance (AMR) genes, integrons, transposases and plasmids found in *Aeromonas hydrophila*, *Aeromonas caviae* and *Aeromonas veronii* isolated from Indian major carp (*Catla catla)*, Indian carp (*Labeo rohita)*, catfish (*Clarias batrachus*) and Nile tilapia (*Oreochromis niloticus*) sampled in India. To gain a wider comparison, we included 11 whole genome sequences of *Aeromonas* spp. from different host species in India deposited in the National Center for Biotechnology Information (NCBI). Our findings show that all 15 *Aeromonas* sequences examined had multiple AMR genes of which the Ambler classes B, C and D β-lactamase genes were the most dominant. The high similarity of AMR genes in the *Aeromonas* sequences obtained from different host species point to interspecies transmission of AMR genes. Our findings also show that all *Aeromonas* sequences examined encoded several multidrug efflux-pump proteins. As for genes linked to mobile genetic elements (MBE), only the class I integrase was detected from two fish isolates, while all transposases detected belonged to the insertion sequence (IS) family. Only seven of the 15 *Aeromonas* sequences examined had plasmids and none of the plasmids encoded AMR genes. In summary, our findings show that *Aeromonas* spp. isolated from different host species in India carry multiple AMR genes. Thus, we advocate that the control of AMR caused by *Aeromonas* spp. in India should be based on a One Health approach.

## Introduction

Aeromonads are Gram-negative facultative anaerobic bacteria ubiquitously found in freshwater, estuarine, and brackish water environments (Janda and Abbott, 2010). Common disease-causing *Aeromonas* species include *Aeromonas hydrophila, Aeromonas caviae*, *Aeromonas veronii*, *Aeromonas sobria* and *Aeromonas salmonicida* (Figueras and Beaz-Hidalgo, 2015). Given their tropism for several species and ubiquitous nature in aquatic environments, *Aeromonas* spp. have the potential to transmit antimicrobial resistance (AMR) genes to multiple host species. Moreover, various *Aeromonas* spp. have been reported to carry plasmids, transposons and integrases that play a major role in acquisition and transfer of AMR genes among different bacteria species (Chang and Bolton, 1987; Sørum et al., 2003; Palu et al., 2006). Thus, a comparison of AMR genes, plasmids, and transposons found in *Aeromonas* spp. isolated from aquatic environments, insects, fish, and animals would shed insight into the role of *Aeromonas* spp. in the spread of AMR genes from the environment to different host species. Information from these studies would guide the design of effective control measures to limit AMR spread by *Aeromonas* spp. in different ecosystems.

India is the second largest consumer of antibiotics after China (Schar et al., 2020). It is also the second largest producer of farmed aquatic organisms in the world (Jayasankar, 2018). Antimicrobials may be used in aquaculture in India for the control of infectious diseases (Walia et al., 2019; Lulijwa et al., 2020) of which *Aeromonas* spp. are among the top pathogens infecting aquatic organisms (Harikrishnan and Balasundaram, 2005; Elgendy et al., 2017; Dubey et al., 2021; Saharia et al., 2021). Boeckel et al. (Van Boeckel et al., 2019) reported that India, together with China, represent the largest environmental AMR hot-spots suggesting that bacteria species like *Aeromonas* spp. ubiquitously found in the aquatic environment are likely to be among the top carriers of AMR genes. *Aeromonas* spp. have been isolated from sewage (Sudheer Khan et al., 2011; Gogry and Siddiqui, 2019), ponds (Singh et al., 2008; Zdanowicz et al., 2020), rivers (Roy et al., 2013), lakes (Joshi, 2016) and marine areas (Vivekanandhan et al., 2005). From farmed aquatic organisms they have been isolated from fresh water loach (*Lepidocephalichthys guntea*) (Roy and Barat, 2011; Roy et al., 2013), freshwater prawn (*Macrobrachium rosenbergii*) (Lijon et al., 2015), marine prawn (*Penaeus semisulcatus*) (Vivekanandhan et al., 2005), Indian white shrimp (*Penaeus indicus*) (Rahimi and Nene, 2006), and giant tiger prawn (*Penaeus monodon*) (Vaseeharan et al., 2005). In insects, they have been isolated from mosquitos (*Culex quinquefasciatus* and *Aedes aegyptii*) (Pidiyar et al., 2002) and chironomid larvae (Kuncham et al., 2017), while from birds and mammals they have been isolated from chickens (Praveen et al., 2014), pigs (Rahimi and Nene, 2006) and buffalo (Rahimi and Nene, 2006). In humans, they have been linked to keratitis, meningitis, and acute gastroenteritis (Misra et al., 1989; Seetha et al., 2004; Sinha et al., 2004; Subashkumar et al., 2006; Motukupally et al., 2014). Overall, these observations from *Aeromonas* studies performed in India are in line with findings from other countries where *Aeromonas* spp. have been isolated from various insect species such as mosquitoes, midges, and houseflies near water bodies (Smith et al., 1998; Pidiyar et al., 2002; Nayduch et al., 2005; Jazayeri et al., 2011) as well as from fish, frogs, reptiles, birds, and mammals (Parker and Shaw, 2011; Elgendy et al., 2017; Wamala et al., 2018; Abdelsalam et al., 2021). What has not been determined is whether *Aeromonas* spp. isolated from different host species carry similar AMR genes, transposons and plasmids.

From previous studies done in India, the prevalence of AMR genes in different *Aeromonas* spp. has been reported using the disc diffusion test and AMR genes by PCR (Sinha et al., 2004; Kaskhedikar and Chhabra, 2010; Roy et al., 2013). A major limiting factor with PCR as a survey tool is that it uses primers targeting only the selected AMR genes, posing the danger of omitting other vital genes contributing to AMR present in bacteria genomes. Thus, PCR lacks the ability to profile all AMR genes present in bacteria genomes. On the other hand, the disc diffusion test only gives the phenotypic characterization of AMR, but does not profile all genes responsible for the antimicrobial resistance. So, the purpose of this study was to use whole genome sequencing (WGS) to identify and compare AMR genes found in *A*. *hydrophila*, *A*. *veronii* and *A*. *caviae* isolated from different fish species in India. To increase our breadth of comparison, we included other publicly available whole genome sequences of *Aeromonas* spp. obtained from different host species in India deposited in the National Biotechnology Center for Information (NCBI). Thus, our work provides a comprehensive overview of AMR genes, efflux pump genes, integrases, transposases and plasmids found in different *Aeromonas* spp. isolated from different host species in India. Data generated herein is useful for creating a basis for a One Health approach in the control of AMR caused by *Aeromonas* spp.

## Materials and methods

### Characterization of bacteria using MALDI-TOF and PCR using 16S rRNA

Two *A. hydrophila* strains (SD/21–01 and SD/21–05) isolated from *Catla catla* and *Labeo rohita*, one *A. veronii* strain (SD/21–04) isolated from *Clarias batrachus* and one *A. caviae* strain (SD/21–11) from *Oreochromis niloticus* from India (Table 1) were retrieved from the −80°C freezer in tryptose soy broth (TSB) and incubated at 30°C overnight. All four *Aeromonas* spp. used were isolated from disease outbreaks of fish cultured under intensive farming (Table 1; Dubey et al., 2021). Diseased fish were treated with oxytetracycline, trimethoprim and sulfonamide. Bacteria grown in TSB were also cultured on blood agar plates for individual colony purity. Purified colonies were further characterized using the Matrix-Assisted Laser Desorption/Ionization-Time Of Flight (MALDI-TOF) mass spectrometry (MS) based on manufacturer’s protocol (Singhal et al., 2015). Purified bacteria confirmed by MALDI-TOF were used for DNA extraction using the DNA extraction kit (Qiagen, Germany). Genus identification was carried out by PCR using universal 16S *rRNA* gene primers 27F and 1492R (Alcock et al., 2020). After confirmation as *Aeromonas* spp. by 16S *rRNA* gene sequencing, cultured isolates were used for genomic DNA extraction.

**Table 1:** Genebank accession numbers of *Aeromonas* spp. used in the study.

Strain	Year	Bacteria species	Host species	Clinical history	Accession no	Source/References
SD/21–04 (Ah2)	2009	*A. veronii*	Walking catfish (*Clarias batrachus*)	Diseased fish	JAJVCV000000000	This study
SD/21–01 (Ah1536)	2009	*A. hydrophila*	Indian carp (*Catla catla*)	Diseased fish	JAJVCT000000000	This study
SD/21–05 (Ah4)	2009	*A. hydrophila*	Rohu *(Labeo rohita)*	Diseased fish	JAJVCU000000000	This study
SD/21–11 (Ah27)	2009	*A. caviae*	Nile tilapia (*Oreochromis niloticus*)	Diseased fish	JAJVCW000000000	This study
XhG1.2	2017	*A. veronii*	Green swordtail (*Xiphophorus hellerii*)	Diseased fish	JACGXR000000000.1	Das et al. (2021)
A8-AHP	2016	*A. veronii*	Rohu *(Labeo rohita)*	Diseased fish	CP046407.1	Tyagi et al. (2022)
Phln2	2010	A. veronii	Fish intestine	Unknown	ANNT00000000.1	
F2S2–1	2015	*A. dhakensis*	Indian oil sardine (*Sardinella longiceps*)	Not specified	LZFM00000000.1	Nadiga et al. (2016)
Y557	2015	*A. salmonicida*	Bighead carp (*Aristichthys nobilis*)	Market foods	JZTH00000000.1	Vincent et al. (2016)
Y567	2015	*A. salmonicida*	Buffer catfish (*Ompok bimaculatus*)	Market foods	JZTG00000000.1	Vincent et al. (2016)
A527	2007	*A. salmonicida*	Giant river prawn (*Macrobrachium rosenbergii*)	Market foods	CP022550.1	Vincent et al. (2017)
CMF	2019	*A. veronii*	Insect gut (***Chrysomya megacephala***)	Unknown	WVRP00000000.1	
FC951	2017	*A. veronii*	Human (*Homo sapiens*)	Asymptomatic patients	CP032839.1	Ragupathi et al. (2020)
VBF557	2015	*A. veronii*	Human (*Homo sapiens*)	Unknown	LXJN00000000.1	
Y47	2015	*A. salmonicida*	Chicken (*Gallus domesticus*)	Market foods	JZTF00000000.1	Vincent et al. (2016)

### Testing of antimicrobial resistance using disk diffusion assay

The four *Aeromonas* spp. isolated from different fish species (Table 1) were tested for antibiotic resistance using the Kirby-Bauer disk diffusion assay (Joseph et al., 2011). Commercially available antibiotic discs (Neo-Sensitabs™, Rosco) used were ampicillin (AMP-10 μg), cefoxitin (CFO-30 μg), cephalothin (CEP-30 μg), ciprofloxacin (CIPR-5 μg), erythromycin (Ery-15 μg), gentamycin (GEN-10 μg), nitrofurantoin (NI-300 μg), penicillin (PEN-10 μg), sulfonamide (SULFA-240 μg), tetracycline (TET-30 μg), and trimethoprim (TRIM-5 μg). Overnight grown bacterial isolates were diluted to 0.5 MacFarland at a concentration of 10^8^cfu/ml and 100 μl spread over the Muller Hinton agar using sterile cotton swabs (Saffari et al., 2016). Antibiotic discs were placed on the agar plate surface on a bacterial lawn followed by incubation at 30°C overnight. Antibiotic susceptibility/resistance was measured based on the manufacturer’s instruction (Neo-Sensitabs™, Rosco Diagnostica, Albertslund, Denmark). All experiments were carried out based on the Clinical and Laboratory Standards Institute (CLSI) (Cockerill et al., 2012) guidelines to determine the susceptibility or resistance of bacteria to antibiotic treatment (Kahlmeter et al., 2006).

### Bacterial genomic DNA extraction and QC analysis

Genomic DNA (gDNA) was extracted from the four *Aeromonas* spp. isolated from fish in India using the MagAttract® HMW DNA kit based on the manufacturer’s protocols (Qiagen GmbH, Hilden, Germany) (Becker et al., 2016). A 1 ml volume containing approximately 2 ×10^9^ CFU/ml freshly grown bacteria was centrifuged in 2 ml Eppendorf tubes and pellets were resuspended in 180 μl buffer ATL (tissue lysis buffer, Qiagen GmbH, Hilden, Germany). Thereafter, Proteinase K (20 mg/ml concentration) was added to each tube followed by incubation at 56°C in an Eppendorf thermomixer for 30 min. After incubation, 4 μl RNase was added to the suspension followed by pulse vortexing. This was followed by adding 15 μl of MagAttract Suspension G and 280 μl Buffer MB to each vial followed by pulse vortexing (Tarumoto et al., 2017). The suspension from each tube was transferred onto the MagAttract holder followed by mixing for 1 min on an Eppendorf thermomixer. Magnetic beads containing gDNA were separated on the MagAttract magnetic rack for around 1 min, and supernatants were removed without disturbing the beads. Magnetic beads were washed twice using MW1 and PE buffer (Becker et al., 2016; Tarumoto et al., 2017). The remaining suspension from each vial was removed by rinsing the beads with 1 ml RNase-free water twice (Qiagen GmbH, Hilden, Germany) (Becker et al., 2016). The harvested gDNA was eluted in 100 μl buffer EB. The purity of gDNA was assessed using the NanoDrop (Thermo Fisher, Arbor, Michigan United States) and gel electrophoresis using 1% agarose. Quantification of gDNA was done using the Qubit double-stranded DNA high-CHS kit based on the manufacturer’s instructions (Life Technologies Inc., Carlsbad, CA, United States) (Guan et al., 2020).

### Library preparation, sequencing and bioinformatic analysis

*Aeromonas* spp. sequence libraries were prepared using the paired-end genome libraries using the Nextera DNA Flex Tagmentation (Illumina Inc. San Diego, CA, United States) (Gaio et al., 2021). Illumina libraries were quantified using the Qubit® DNA HS Assay Kit in a Qubit fluorometer (Thermo Fisher Scientific, Waltham, MA, United States) while the size of library fragments was checked using an Agilent 2,100 Bioanalyzer System using the Agilent HS DNA Kit (Agilent Technologies, CA, United States). Illumina MiSeq (Illumina Inc., United States) were sequenced using V3 reagent kits using paired-end read length of 2 × 300 bp (Kaspersen et al., 2020). Four bacterial raw DNA reads from this study and 11 sequence reads archives (SRAs) were retrieved from NCBI (Table 1) and were analyzed using the online Galaxy platform (https://usegalaxy.no/) version 21.05. Quality of both forward and reverse raw reads were analyzed using the FastQC Version 0.11.9 software (Bioinformatics, 2011), while the Trimmometric version 0.38.1 was used to remove the adapters and low-quality reads from paired-end sequences (Bolger et al., 2014). The resulting paired-end sequence reads were *de novo* assembled into contigs using SPAdes v. 3.12.0 (Coil et al., 2015) with 33 to 91 k-mers (Bankevich et al., 2012), while genome annotation was conducted using the prokaryotic genome annotation pipeline (PGAP) (Tatusova et al., 2016) from the NCBI and Prokka (Seemann, 2014).

### Prediction of antimicrobial resistance genes

In addition to genome sequences of the four isolates from fish in India, we retrieved 11 whole genome sequences (WGS) of *Aeromonas* spp. from different host species in India from the NCBI database for comparison with our isolates (Table 1). Among the retrieved genomes from NCBI, *A. veronii* strain A8-AHP was isolated from the kidney tissue of diseased *Labeo rohita* and was shown to have reduced susceptibility for ampicillin and imipenem on the disk diffusion test (Tyagi et al., 2022), while *A. veronii* strain XhG1.2 was isolated from gills and intestine of diseased green swordtail fish and no antibiotic resistance test was reported (Das et al., 2020). Other *A. veronii* isolates include strain FC951 isolated from healthy humans, VBF557 from humans with unknown clinical history, CMF from insect gut (*Chrysomya megacephala*) and PhIn2 from fish intestines with unknown clinical history (Table 1). Similarly, *A. dhakensis* strain F2S2–1 was isolated from the skin surface of an Indian oil sardine (Nadiga et al., 2016). The *A. salmonicida* strains Y47, Y567, A527 and Y577 were isolated from a chicken, butter catfish (*Ompok bimaculatus*), prawn (*Macrobrachium rosenbergii*), and bighead carp (*Aristichthys nobilis*), respectively, sold as food at a market in Mumbai in India (Nagar et al., 2011; Vincent et al., 2017). There was no information available regarding the antibiotic treatment of the host species for the genomes retrieved from the NCBI database and no record of disc diffusion test for all isolates, except *A. veronii* strain A8-AHP. Altogether, a total of 15 sequences were used for WGS comparison of AMR genes, plasmids and transposases profiles. Antibiotic resistance genes were identified using staramr version 0.7.2 (Tran et al., 2021) and ABRicate version 1.0.1 (Seemann, 2016) in the Comprehensive Antimicrobial Resistance Database (CARD) (Alcock et al., 2020). The threshold for AMR gene identification using the CARD was set at 80%. Plasmidfinder v 2.0 (Ullah et al., 2020) was used to identify plasmids in the bacterial genomes.

### Pangenome analysis

The pangenome of the 15 *Aeromonas* isolates from India was constructed using Roary version 3.13.0 using general feature files 3 (.gff) generated from Prokka Version 1.14.5. The minimum percent identity cut-off limit was set at 95% (Seemann, 2014; Page et al., 2015). The distribution of core genes (genes present in all genomes), shell genes (genes not shared by all genomes but present in more than one isolate), and cloud genes (genes only found in one isolate) were determined using the online usegalaxy.no platform with minimum gene identity cut-off of 99% (Page et al., 2015). The pangenome of all 15 *Aeromonas* genomes was generated using the online genome viewer Phandango (Hadfield et al., 2018), while accompanying phylogenetic trees were created using Gene_presence_absence and Newick files obtained from Roary and *Aeromonas* genomes were grouped in similarity clusters.

### Phylogenetic analysis of antimicrobial resistance genes

Phylogenetic analysis of the Ambler classes B, C and D β-lactamase genes was carried out using the Molecular Evolutionary Genetic Analysis version 7 (MEGA-7) bioinformatics software (Kumar et al., 2016). The AMR genes used for phylogenetic analyses were retrieved after screening of AMR genes using ABRicate version 1.0.1 for all 15 *Aeromonas* spp. genomes. Phylogenetic trees were generated using the Neighbor-joining and BioNJ algorithm to a pairwise matrix estimated using JTT model and expressed as number of base substitution per site (Jones et al., 1992). The outlier groups for the Ambler classes B, and C β lactamase genes used were *Shigella sonnei* tetracycline gene *tet(A)* ANN06707.1 while *Vibrio fluvialis* sulfonamide gene *sul1* AEJ33969.1 was used as out group for the class D β-lactamase and the *CRP* gene, respectively.

## Results

### Phenotype characterization of antimicrobial resistance using the disc diffusion test

All four *Aeromonas* spp. isolated from fish in India showed multidrug resistance (MDR) to three or more antibiotics on the disk diffusion test (Table 2). *A. hydrophila* strain SD/21–01 from Indian carp (*C. catla*) was resistant to AMP-10, CEP-30, PEN-10, ERY-15, SULFA-240, while the *A. hydrophila* strain SD/21–05 (*L. rohita*) was also resistant to AMP-10, ERY-15, PEN-10, SULFA-240 and TRIM-5. The *A. veronii* isolate from catfish (SD/21–04) was resistant to AMP-10, CFO-30, CEP-30, ERY-15, GEN-10, PEN-10, and TET-30 while *A. caviae* from Nile tilapia (SD/21–11) was resistant to AMP-10, PEN-10, ERY-15, GEN-10 and SULFA-240. All four isolates were susceptible to CIPR, and NI300. In addition, *A. hydrophila* from India carp (SD/21–05) and catfish (SD/21–01) together with *A. caviae* from Nile tilapia (SD/21–11) showed susceptibility to TET-30 while *A. veronii* from catfish (SD/21–04) was susceptible to SULFA-240.

**Table 2:** Antibiotic susceptibility of *Aeromonas* spp. based on disk diffusion test.

Antibiotics (μg)	SD/21–04	SD/21–01	SD/21–05	SD/21–11
Ampicillin (AMP-10)	R	R	R	R
Cefoxitin (CFO-30)	R	R	S	S
Cephalothin (CEP-30)	R	R	S	S
Ciprofloxacin (CIPR-5)	S	I	S	I
Erythromycin (ERY-15)	R	R	R	R
Gentamycin (GEN-10)	R	R	I	R
Nitrofurantoin (NI-300)	S	S	S	S
Penicillin (PEN-10)	R	R	R	R
Sulfonamide (SULFA-240)	R	R	R	R
Tetracycline (TET-30)	R	I	I	S
Trimethoprim (TRIM-5)	I	I	R	I

Determination of susceptibility or resistance to antibiotic treatment was based on the Clinical and Laboratory Standards Institute (CLSI) guidelines (Kahlmeter et al., 2006; Cockerill et al., 2012).

### Genome comparison

Draft genomes of all the four *Aeromonas* isolates from India sequenced using the MiSeq 300 generated varied between 44.5–52.0 million DNA reads with a phred quality score > 36 for all four isolates (Table 3). After quality filter (Q > 30), approximately 42.6–44.3 million reads were *de novo* assembled using SPAdes v. 3.12.0. Raw data generated after sequencing have been deposited in NCBI under the sequence read archive (SRA) accession numbers from SRR17405115 to SRR17405118. Genome assembly and annotation features of the four fish isolates together with 11 genomes from other species are shown in Table 3. Final genome assembly of the four Indian fish isolates SD/21–01, SD/21–05, SD/21–04 and SD/21–11 consisted of 4,701,638 bp, 4,940,355 bp, 4,570,779 bp, 4,231,844 bp, with N50 value 766,346 bp, 239,795 bp, 184,893 bp, 101,699 bp, respectively. Total number of contigs for *A. hydrophila* (SD/21–01 and SD/21–05), *A. veronii* (SD/21–04) and *A. caviae* (SD/21–14) were 30, 78, 69 and 99, respectively. All four fish genomes have been deposited at DDBJ/ENA/GenBank with accession JAJVCT000000000 to JAJVCW000000000 (Table 1). The size of all 15 genomes is shown in Table 3. Equally, a comparison of other parameters such as contigs, G + C content %, genes (total), genes (RNA), protein coding genes (CDS), and Pseudo Genes is shown in Table 3.

**Table 3:** Whole genome sequence data of *Aeromonas* spp. used in the study.

Genome feature	SD/21–04	SD/21–01	SD/21–05	SD/21–11	XhG1.2	A8-AHP	PhIn2	F2S2–1	Y557	Y 567	A527	CMF	FC951	VBF557	Y47
**Source**	Fish	Fish	Fish	Fish	Fish	Fish	Fish	Fish	Fish	Fish	Prawn	Insect gut	Human	Human	Chicken
**Country of origin**	India	India	India	India	India	India	India	India	India	India	India	India	India	India	India
**Contig**	69	30	78	99	35	1	1899	64	104	47	1	200	1	526	118
**Genome size (bp)**	4,570,779	4,701,638	4,940,355	4,231,844	4,573,855	4,744,657	4,300,552	4,750,839	4,736,406	4,554,843	4,806,250	4,562,071	4,666,657	4,696,503	4,710,230
**Largest contig**	602,701	1,325,364	610,305	352,808	-	4,744,657	23,680	-	-	-	4,806,250	-	4,666,657	-	-
**N50**	184,893	766,346	239,795	101,699	305,294		3,789	260,482	101,766	217,341		40,276	-	19,666	117,778
**G + C content %**	58.60	61.26	60.92	62.02	58.7	58.36	58.68	60.30	62.05	59.67	61.87	56.7	60.50	61.53	62,5
**Genes (total)**	4,310.0	4,361	4,613	3,993	4,165	4,419	3,993	4,225	4,389	4,215	4,470	4,282	4,575	4,460.0	4,400.0
**CDSs (with protein)**	4,152	4,203	4,425	3,822	4,038	4,205	3,822	4,075	4,154	4,007	4,138	4,143	4,056	3,325	4,165
**Genes (RNA)**	109	109	108	101	78	145	101	115	146	147	162	96	147	93	154
**rRNAs**	4	5	4	5	4	31	5	21	31	33	31	11	21	15	35
**tRNAs**	100	97	96	90	70	108	90	88	115	114	125	81	112	74	119
**Pseudo Genes (total)**	49	49	80	70	53	69	70	35	88	57	170	43	372	1,042	78

### Pangenome analysis

**Figure 1:**
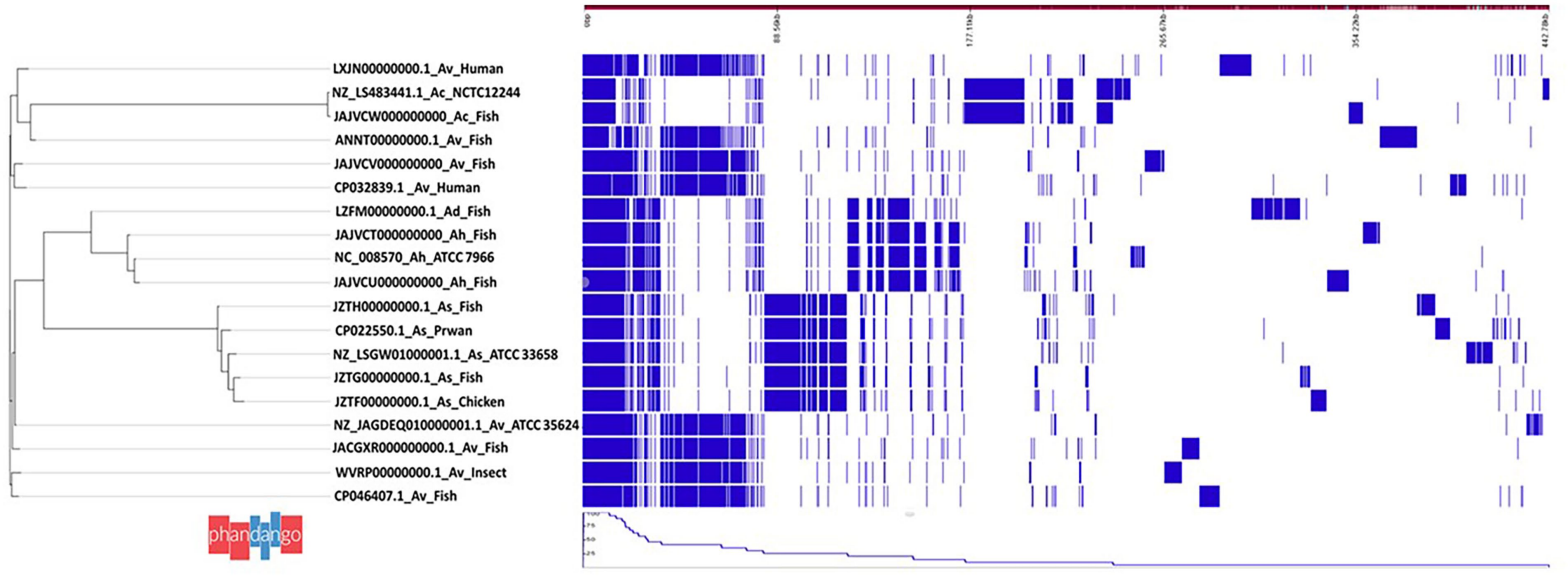
Pangenome analysis of 15 *Aeromonas* spp. isolated from different host species in India. Note that the 15 *Aeromonas* spp. are in four groups based on species. (i) Group comprises of *Aeromonas veronii* isolates and reference strain NZ_LS4883441.1 NCTC12244, (ii) Group 2 consists of a *A. hydrophila* and *A. veronii* isolate and reference strain NC_008570 Ah_ATCC7966, (iii) Group 3 consists of *A. salmonicida* isolates and reference strain NZ_LSGW01000001.1_As_ATCC33658, and (iv) Group 4 consists of *A. hydrophila* and *A. dhakensis* isolates together with the NZ_JAGDE01000001.1 Av_ATCC35624 reference strain.

The total number of genes detected from the 15 *Aeromonas* genomes (Table 1) based on pangenome analysis was 20,415 genes of which 621 genes were core-, 7,139 shell- and 12,655 cloud genes (Figure 1). Four groups were generated based on *Aeromonas* species classification. Group 1 consisted of seven *A. veronii* genomes obtained from catfish (SD/21–04), human (FC951 and VBF557), fish (Ph1n2), Indian carp (A8-AHP), swordtail (XhG1.2) and insect (CMF). The total number of genes from group-1 was 8,911 genes that comprised of 2,388 core-, 1898 shell- and 4,625 cloud genes. Group-2 only comprised of genes from *A. caviae* isolated from Nile tilapia (SD/21–11). Group-3 consisted of genes from four *A. salmonicida* genomes from prawn (A527), butter catfish (Y567), chicken (Y47) and bighead carp (Y557) that had a total of 6,048 genes comprising of 3,320 core and 2,728 shell genes. Group-4 consisted of genes from two *A. hydrophila* genomes isolated from Indian carp (SD/21–01 and SD/21–05) and one *A. dhakensis* genome from sardine (F2S2–1). The total number of genes in group-4 was 6,321 genes of which 2,786 were core- and 3,535 shell genes.

### Antibiotic resistance genes

All 15 *Aeromonas* genomes analyzed had three or more AMR genes of which the Ambler classes B, C and D β-lactam genes accounted for the majority (Table 4).

**Table 4:** Antimicrobial resistance genes detected in the *Aeromonas* genomes.

AMR gene description	Gene	SD/21-04	SD/21-01	SD/21-05	SD/21-11	XhG1.2	A8-AHP	Phln2	F2S2-1	Y557	Y567	A527	CMF	FC951	VBF557	Y47
Class A β-lactamase	*TEM-150*															
Ambler Class B MBL (Carbapenem)	*ImiH*															
	*cphA4*															
	*cphA3*															
	*cphA5*															
	*cphA8*															
Class C beta-lactamase (Cephalosporin Cephamycin Penam)	*Aqu-2*															
	*CepS_*															
	*bla*_MOX-7_															
	*bla*_FOX-7_															
	*bla*_FOX-4_															
	*bla*_FOX-2_															
	*bla*_FOX-5_															
Class D β-lactamase (Cephalosporin, Penam)	*bla*_OXA-12_															
	*bla*_OXA-427_															
	*bla*_OXA-724_															
	*bla*_OXA-780_															
Trimethoprim-resistant	*dfrA12*															
Aminoglycoside	*aadA2*															
	*APH(3′)-Ia*															
Sulfonamide resistant	*sul1*															
Aaminoglycoside	*ANT(3″)-IIa*															
Total resistance genes	3	3	6	3	3	3	3	3	3	3	3	3	3	3	3	

All antimicrobial resistance genes detected and identified using staramr version 0.7.2 (Tran et al., 2021) and ABRicate version 1.0.1 (Seemann, 2016) in the Comprehensive Antimicrobial Resistance Database (CARD) (Alcock et al., 2020).

Blue = presence of resistance genes (detected), White/blank = absence of gene (not detected).

#### Ambler class B metallo-β-lactam resistance genes

**Figure 2:**
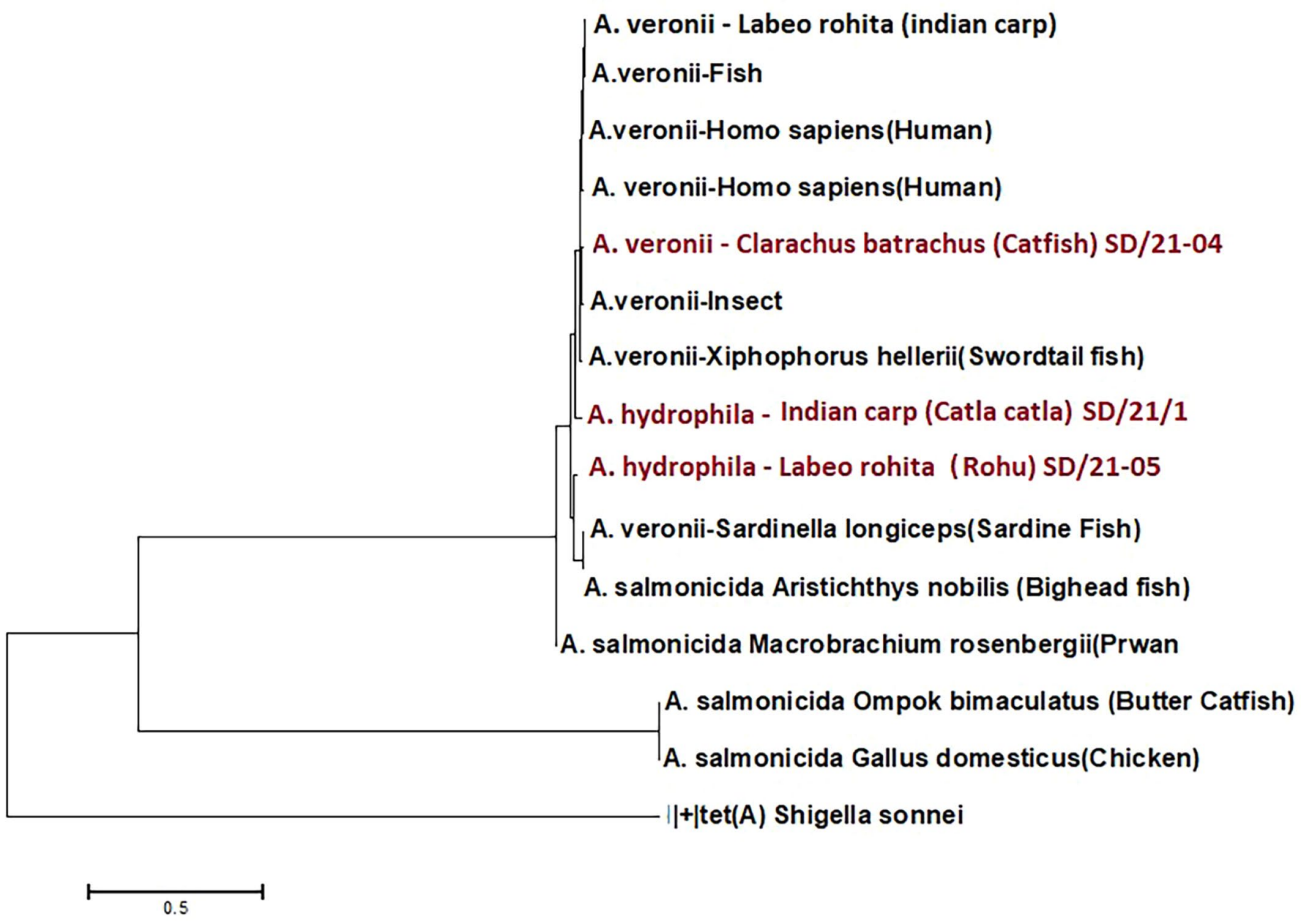
Phylogenetic analysis of Ambler class B metallo-β-lactam (MBL) resistance genes from 13 *Aeromonas* spp. isolated from different host species in India.

Among the Ambler class B metallo-β-lactamase (MBL) resistance genes, only five AMR genes were detected from 14 of the 15 *Aeromonas* genomes examined (Table 4) comprising of (i) carbapenem gene *ImiH* from *A. hydrophila* isolated from Indian carp (SD/21–01), (ii) carbapenem gene *cphA3* from *A. veronii* isolated from humans (FC951 and VBF-557), Swordtail fish (XhG1.2), Indian carp (A8-AHP), and *A. dhakensis* from sardine (F2S2–1), (iii) carbapenem gene *cphA4* from *A. veronii* from catfish (SD/21–04), insect gut (CMF) and fish (PhIp2), (vi) carbapenem gene *cph5* detected from *A. salmonicida* isolated from chicken (Y47), prawn (A527), butterfish (Y567) bighead fish (Y557), and (v) carbapenem gene *cphA8* from *A. hydrophila* isolated from Indian carp (SD/21–05). Phylogenetic analysis showed a close similarity for all Ambler class B MBL genes genes isolated from different *Aeromonas* spp. in spite of the bacteria isolates coming from different host species (Figure 2).

#### Class C β-lactamase resistance genes

**Figure 3:**
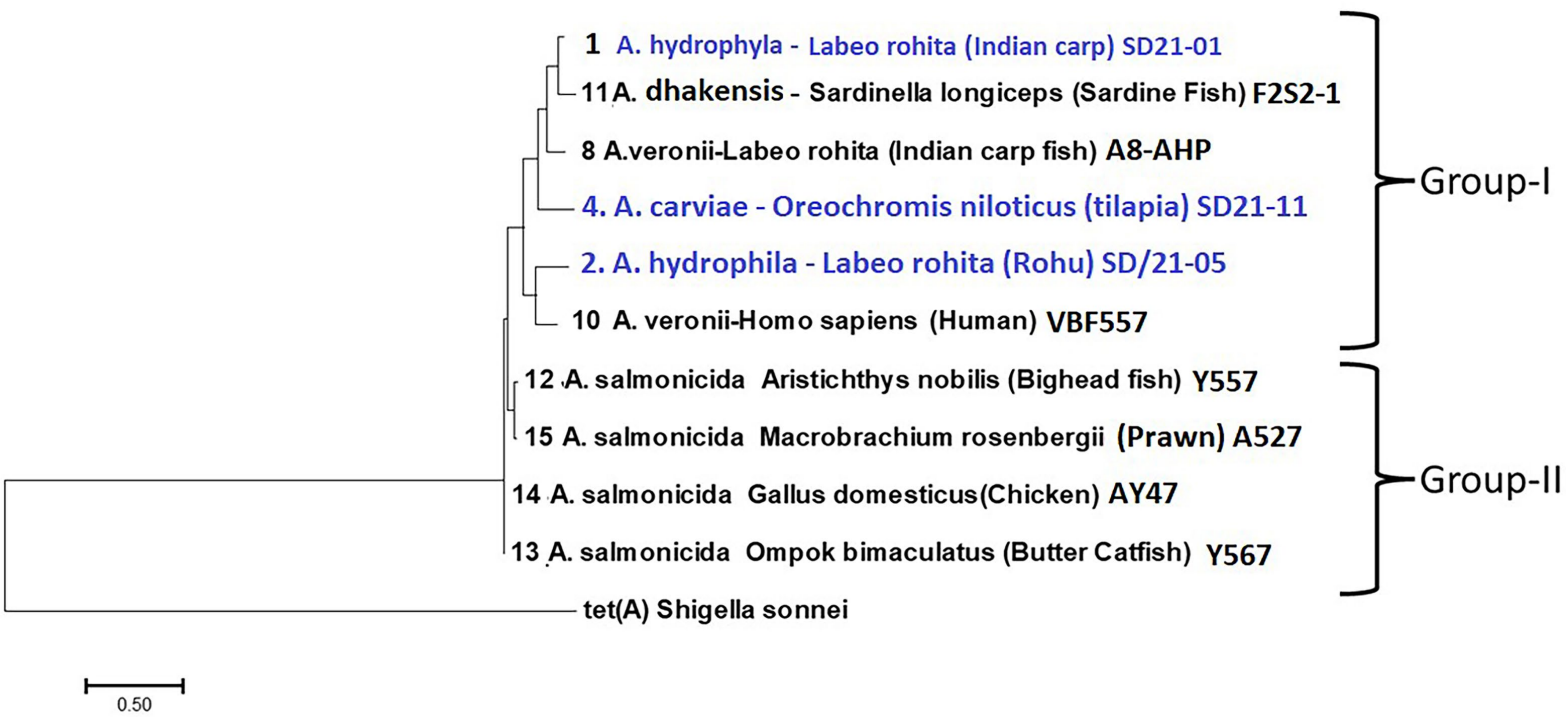
Phylogenetic analysis of Ambler class C β-lactam resistance genes from 10 *Aeromonas* spp. isolated from different host species in India. The isolates were put in two groups. (i) Group 1 consists of *bla*_AQU-2_, *cepS*, *bla*_MOX-7_, and *bla*_FOX-7_ genes from *A. hydrophila*, *A. veronii*, and *A. caviae* isolated from different host species. Note that Strains SD/21–01 and F2S2/1 having the *bla*_AQU-2_ gene were put together while strains SD/21–05 and VBF557 having the *cepS* gene were also placed together. (ii) Group 2 consists of *bla*_FOX-2_, *bla*_FOX-4_ and *bla*_FOX-5_ genes from *A. salmonicida* isolates of which strains Y567 and Y527 having the *bla*_FOX-2_ gene were put together.

**Figure 4:**
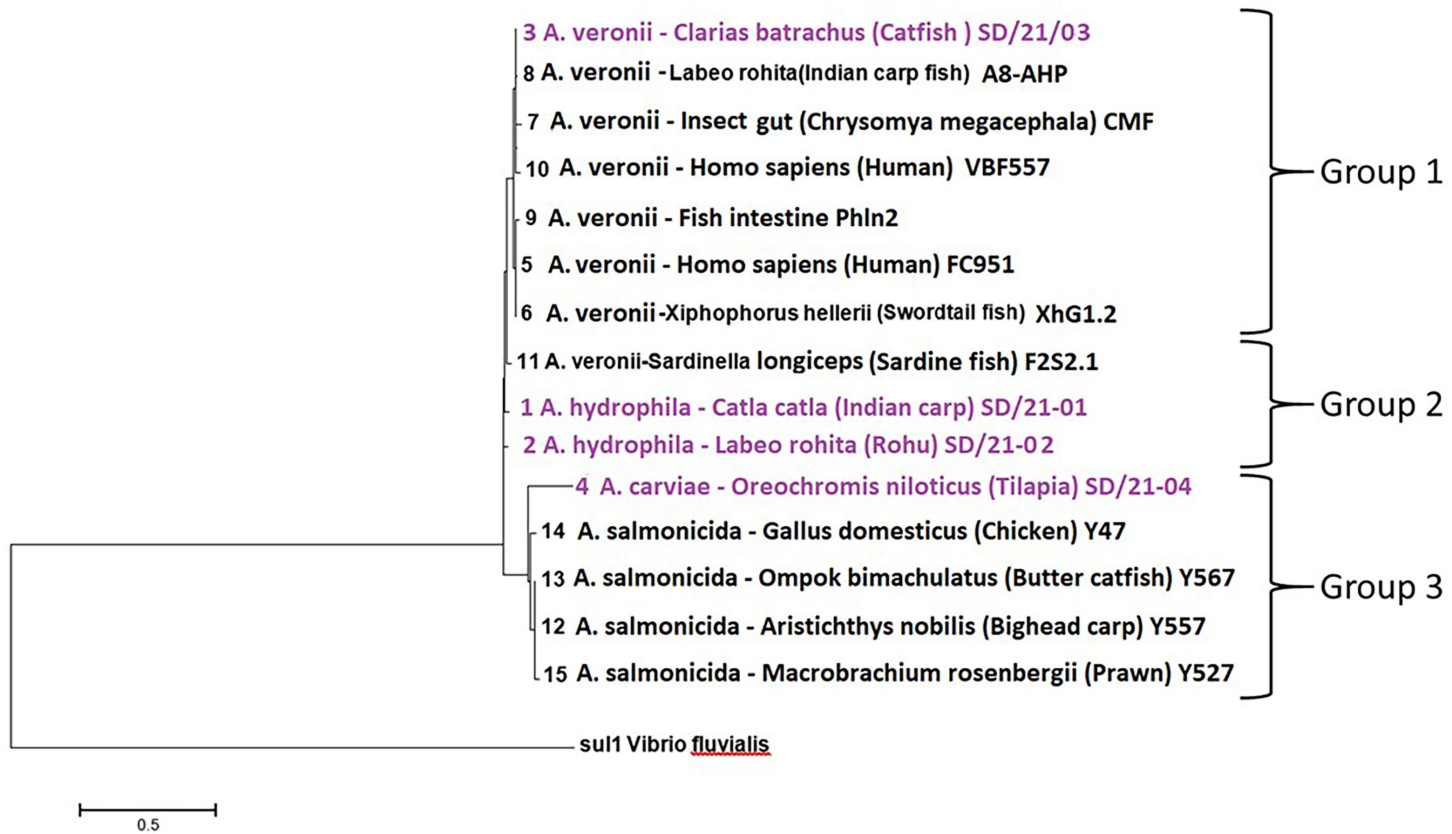
Phylogenetic analysis of D class β-lactam resistance genes from 15 *Aeromonas* spp. from different host species in India. (i) Group 1 consists of the *bla*_OXA-12_ gene from *A. veronii* isolates, (ii) Group 1 had the *bla*_OXA-724_ gene from *A. veronii*, and *A*. *hydrophila* isolates while (iii) Group 3 has the *bla*_OXA-427_ gene from *A. caviae* and *A. salmonicida* isolates.

Of the 15 aeromonas genomes examined, only nine had class C β-lactam resistance genes (Table 4) consisting of (i) β-lactamase gene *bla*_AQU-2_ from *A. hydrophila* isolated from Indian carp (SD/21–1) and *A. dhakensis* (F2S2–1) from sardine, (ii) cephalosporin gene *cepS* from *A. hydrophila* (SD/21–05) isolated from Indian carp and human (VBF557), (iii) *bla*_MOX-7_ from *A. caviae* isolated from Nile tilapia (SD/21–11), and (iv) *bla*_FOX-7_ from *A. veronii* isolated from Indian carp (A8-APH). Resistance genes detected from *A. salmonicida* isolates included (v) *bla*_FOX-2_ from butter catfish (Y567) and prawn (A527) (vii), *bla*_FOX-4_ from bighead fish (Y557) and *bla*_FOX-5_ from *A. salmonicida* from chicken (Y47). The phylogenetic tree divided the Class C β-lactamase resistance genes in two groups of which group 1 comprised of the *bla*_AQU-2_, *cepS*, *bla*_MOX-7_ and *bla*_FOX-7_ genes while the *bla*_Fox_ genes from *A. salmonicida* were clustered together in group 2 (Figure 3). Phylogenetic analysis showed that *A. hydrophila* strain SD/21–01 from Indian carp and *A. dhakensis* strain F2S2–1 from sardine that had the β-lactamase gene *bla*_AQU-2_ were paired together, while *A. hydrophila* strain SD/21–05 from Indian carp and strain VBF557 from human that also had the cephalosporin gene *cepS* were also put next to each other in group I. Equally, the *bla*_FOX-2_ gene from *A. salmonicida* strains Y567 from butter catfish and A527 from prawn were placed next to each other in group II. Altogether, these findings show that genes identified to be similar using the CARD (Alcock et al., 2020; Table 4) also had a high similarity in the phylogenetic tree (Figure 4). Overall, these findings point to high similarity among C β-lactamase resistance genes in spite of the bacteria isolates coming from different host species (Figure 3).

#### Classes D β-lactamase resistance genes

Only resistance genes belonging to the *bla*_OXA_ group were detected in class D β-lactamase. The first group consisted of *bla*_OXA-12_ from *A. veronii* isolated from catfish (*C. catla*) (SD/21–04) and Swordtail fish (XhG1.2) together with *A. veronii* from humans (FC951 and VBF-557), insect (CMF), Indian carp (A8-AHP), and fish (Phln2) (Table 4). The second group comprised of *bla*_OXA-724_ from *A. hydrophila* isolated from Indian carp (SD/21–01 and SD/21–05) and *A. dhakensis* from sardine (F2S2–1). The third group consists of *bla*_OXA-427_ from *A. salmonicida* isolates from bighead fish (Y557), prawn (A527), chicken (Y47), and butter catfish (Y567). The final group consisted of *bla*_OXA-780_ from *A. caviae* isolated from Nile tilapia (SD/21–11). The phylogenetic tree showed that all seven isolates having the *bla*_OXA-12_ had 100% similarity comprising of *A. veronii* from catfish (*C. catla*) (SD/21–04), Indian carp (A8-APH), insect (CMF), human (FC951), swordtail (XhG1.2), fish intestine (Ph1n2) and human (VBF557) clustered together in group 1 (Figure 4). Equally, isolates that had the *bla*_OXA-724_ gene inclusive of *A. hydrophila* isolated from Indian carp (SD21/05 and SD21/01) and *A. veronii* from sardine (F2S2–1) had a 100% similarity and were clustered in group 2 while the *bla*_OXA-427_ gene detected in four *A. salmonicida* isolates from bighead (Y557), butter catfish (Y567) and prawn (Y47) was associated with group 3 with 100% similarity (Figure 4). Thus, these findings show that the similarity in AMR genes identified based on the CARD (Alcock et al., 2020; Table 4) corresponded with the similarity seen in the phylogenetic tree (Figure 4). Altogether, these findings show high similarity of class D β-lactam resistance genes irrespective of the bacteria being isolated from different host species.

#### Other antibiotic resistance genes

Only *A. hydrophila* isolated from the Indian carp (SD/21–05) possessed the trimethoprim resistance gene *dfrA12* (Table 4) being in agreement with the disk diffusion test results of resistance to trimethoprim (Table 2). The sulfanomide resistance gene *sul1* was only detected from *A. hydrophila* and *A. caviae* isolated from Indian carp (SD/21–05) and Nile tilapia (SD/21–11) (Table 4) that also showed resistance to sulfonamide in the disk diffusion test (Table 2). Aminoglycoside resistance genes comprised of *aadA2* from *A. hydrophila* isolated from Indian carp (SD/21–05), *APH(3′)-Ia* from *A. veronii* isolated from Indian carp (A8-AHP), and *ANT(3″)-IIa* from *A. caviae* isolated from Nile tilapia (SD/21–11). The tetE gene was only detected from *A. veronii* (SD/21–04) isolated from catfish that also showed resistance to tetracycline in the disk diffusion test (Table 2) and *A. salmonicida* from chicken (Y47), while *bla*_TEM-150_ was only detected from *A. veronii* isolated Indian carp (PhIn2). Other genes detected include the chloramphenicol gene *cmlA1* and colistin gene *mrc-3* from *A. veronii* isolated from Nile tilapia (SD/21–11), humans (FC951), and Swordtail fish (XhG1.2), respectively. Correlation of the phenotypic profile determined by the disk diffusion test with the genotypic profile based on genes identified using the CARD (Alcock et al., 2020) showed 93% specificity and 88% sensitivity with an overall kappa score (K) of 0.88 determined using the Cohen’s kappa test (Cohen, 1968).

### Drug resistance efflux pump genes

**Figure 5:**
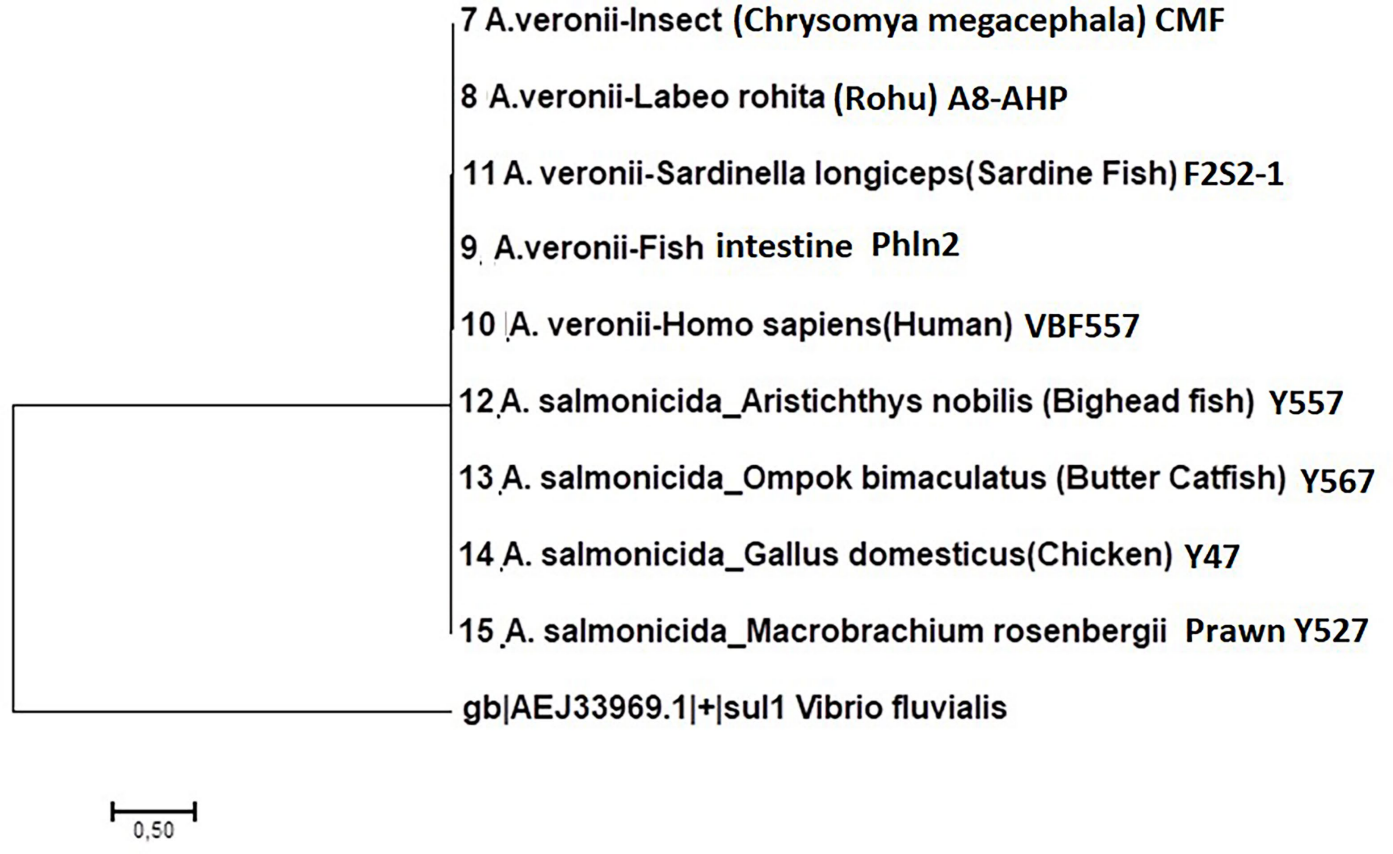
Phylogenetic analysis of *crp* resistance genes detected from sequences of nine *Aeromonas* spp. isolated from different host species in India.

All 15 *Aeromonas* genomes had multiple multidrug efflux pump genes (Table 5). Dominant genes included the multidrug resistance protein (*mdtH*) and the zinc/cadmium/mercury/lead-transporting ATPase *zntA* gene detected in all 15 *Aeromonas* genomes. Other dominant genes included *mdtL*, which was detected in 11 of the 15 *Aeromonas* genomes except for *A. veronii* isolated from catfish (SD/21–04), green swordtail fish (XhG1.2), insect (CMF), and *A. salmonicida* from prawn (A527) (Table 5). The multidrug efflux MFS transporter (*emrD*), β-lactam sensor histidine kinase (*blrB*), and bleomycin resistance family protein (brp) were detected in *A. hydrophila* isolated from Indian carp (SD/21–01 and SD/21–05) and *A. veronii* isolated from catfish (SD/21–04), human (FC951), and insect (CMF) but not from the *A. salmonicida* and *A. dhakensis* (F2S2–1). On the contrary, the emrB/QacA family drug resistance (*emrB*), bicyclomycin resistance protein (*BCM*), Putative chloramphenical resistance permease protein (*rarD*) and fluoroquinolone (*qnr*) were dominant in the *A. salmonicida* and *A. dhakensis* isolates but absent in *A. hydrophila* and less dominant in *A. veronii* isolates. Finally, the resistance nodulation cell division (RND) multidrug efflux pump *crp* was detected from five *A. veronii* isolates from insect (CMF), Indian carp (A8-AHP), fish (PhIn2), and humans (VBF557) as well as *A. dhakensis* from sardine (F2S2–1). In addition, *crp* was also detected from four *A. salmonicida* isolates from bighead fish (Y557), prawn (A527), chicken (Y47), and butter catfish (Y567). Phylogenetic analysis showed a similarity of 100% *crp* from *A. veronii* isolates from insect, Indian carp, and human isolates (Figure 5). The homology among *crp* from the nine host species varied between 99.1 and 100.0%. Other resistance drug efflux genes detected are shown in Table 5.

**Table 5:** Multidrug efflux pump genes detected in *Aeromonas* genomes.

Gene description	Gene	SD/21–04	SD/21–01	SD/21–05	SD/21–11	XhG1.2	A8-AHP	Phln2	F2S2–1	Y557	Y567	A527	CMF	FC951	VBF557	Y47
Multidrug resistance protein (fluoroquinolones, ceftriaxone)	*mdtL*															
Multidrug resistance protein (fluoroquinolone)	*mdtH*															
Multidrug resistance protein (Aminocoumarin)	*mdtB*															
multidrug efflux MFS transporter (Phenicol)	*emrD*															
EmrB/QacA family drug resistance	*emrB*															
multidrug efflux RND transporter (tetracycline, chloramphenicol)	*MDR*															
chloramphenical resistance permease	*rarD*															
fluoroquinolone resistance protein	*FQR*															
Quaternary ammonium efflux SMR transporter	*QacEdelta1*															
Zinc/cadmium/mercury/lead-transporting ATPase	*zntA*															
Beta-lactam sensor histidine kinase	*blrB*															
organic hydroperoxide resistance protein	*ohrP*															
bleomycin resistance family protein	*ble*															
fosfomycin resistance glutathione transferase	*FosA*															
Bicyclomycin resistance protein	*BCM*															
cAMP-activated global transcriptional regulator	*crp*															

All multidrug efflux pump genes were detected and identified using staramr version 0.7.2 (Tran et al., 2021) and ABRicate version 1.0.1 (Seemann, 2016) in the Comprehensive Antimicrobial Resistance Database (CARD) (Alcock et al., 2020).

Blue = presence of genes (detected), White/blank = absence of gene (not detected).

### Resistance genes detected together with integrase and efflux pumps

**Figure 6:**
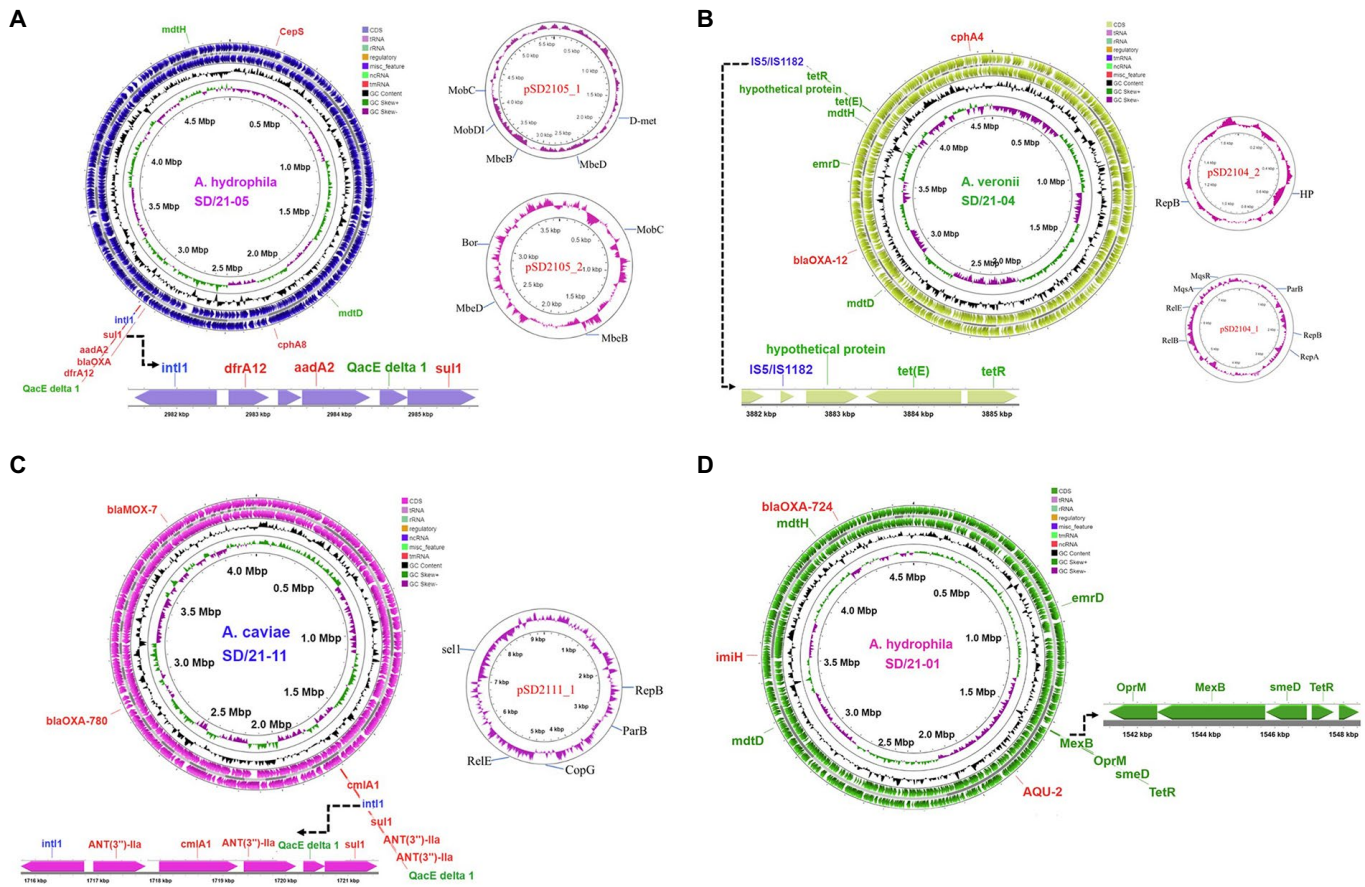
Circular maps of whole genome sequences of *Aeromonas* spp. isolated from four fish species in India. **(A)** Circular map of whole genome sequence of *Aeromonas hydrophila* strain SD21/21–05 (blue) isolated from Indian carp (*Labeo rohita*) showing the loci for the resistance genes (red), efflux pumps (green) and integrase (blue). The extended linear map shows the integrase *intl1* linked to *dfrA12*, *aadA2*, *QacEdelta 1* and *sul1*. Circular maps of the two plasmids detected are shown in pink. **(B)** Circular map of whole genome of *A. veronii* strain SD/21–04 isolated from *Clarias batrachus* showing the loci for resistance genes (red), efflux pumps (green), and transposases (blue). The linear map shows the transposases linked to the hypothetical protein, tet(E) efflux pump and *TetR* is the repressor of the tetracycline resistance element. Circular maps of the two plasmids are shown in pink. **(C)** Circular map of the *A. caviae* strain SD/21–11 isolated from *Oreochromis niloticus* showing positions of the resistance genes (red), integrase (blue) and efflux pumps (green) with the linear map showing the integrase *intl1* linked to the *ANT(3″)-IIa*, *cmlA1*, *ANT(3″)-IIa*, *QacEdelta 1* and *sul1* genes. Circular map of the single plasmid is shown in red. **(D)** Circular map of *A. hydrophila* strain SD/21–01 isolated from *Catla catla* showing resistance genes (red) and efflux pump proteins (green). The extended linear map shows the multidrug complex OprM-MexB and RND pumps linked to the *tetR* gene.

The circular map for *A. hydrophila* strain SD/21–05 (Figure 6A) genome showed presence of all six resistance genes (*bla*_OXA-724_, *cepS*, *cphA8*, *dfrA12*, *aadA2*, and *sul1*) detected using the CARD (Alcock et al., 2020; Table 4). It is noteworthy that the integrase *intl1* gene was located next to the trimethoprim (*dfrA12*), aminoglycoside (*aadA2*), and sulfanomide (*sul1*) genes together with the major facilitator superfamily (MFS) efflux pump *QacEdelta-1* (Figure 6A). The circular map of the *A. veronii* strain SD/21–04 (Figure 6B) genome shows that the *tetR* gene was located next to the Tet(E) efflux pump together with an unknown hypothetical protein while other genes detected included the cephalosporin/penam *cphA4* and *bla*_OXA-12_ genes (Figure 6B). As for *A. caviae* strain SD/21–11, our findings show that all four AMR genes *bla*_OXA-12_, *bla*_OXA-780_, *suI2* and *ANT(3)-IIa* detected using the CARD (Alcock et al., 2020) were found in its genome of which the *intI1* integrase was located next to the sulfonamide *suI2* and aminoglycoside *ANT(3)-IIa* genes together with the chloramphenicol *cmlAI* and MFS *QacEdelta-1* efflux pumps (Figure 6C). Finally, the circular map for *A. hydrophila* strain SD/21–01 showed presence of *lmiH*, *bla*_AQU-2_, *bla*_OXA-724_ and *tet(R)* genes in its genome of which the tetracycline gene *tet(R)* was located next to the multidrug complex MexAB-OprM and the RND smeD efflux pumps.

### Plasmids found in the *Aeromonas* spp.

Of the four *Aeromonas* spp. sequenced in the present study, three had plasmids (Supplementary Table S1). *A. hydrophila* from Indian carp (SD/21–05) had two plasmids of which pSD2105-1 had a size of 5,278 bp while pSD2105-2 was 3,599 bp (Figure 6A). Genes found in pSD2105-1 included *D-met*, *mebB*, *mebD*, and *MobDI*, whereas pSD2105-2 had *mobC*, *mbeB*, *mbeD* and *Bor* genes (Supplementary Table S1; Figure 6A). Equally, *A. veronii* from catfish (SD/21–04) had two plasmids with sizes of 7,480 bp (pSD2104-1) and 1740 bp (pSD2104-2) (Figure 6B). Genes detected in pSD2104-1 were *parB*, *repB*, *relB*, *relE*, *mqsA*, and *mqrR*, whereas pSD2104-2 had *hyp* and *repB* (Supplementary Table S1). *A. caviae* from Nile tilapia (SD/21–11) had only one plasmid (pSD21-11) with a size of 9,364 bp that had *repB*, *parB*, *copG*, *relE* and *sel1* genes (Figure 6C). Suffice to point out that only pSD211-11 had a “site-specific integrase.” Only *A*. *hydrophila* from catfish (SD/21–01) had no plasmid (Figure 6D) out of the four *Aeromonas* spp. sequenced in the present study. Of the 11 *Aeromonas* genomes retrieved from NCBI, only three had plasmids (Supplementary Table S1). *A. veronii* from human (FC9l51) had one plasmid (196,528 bp) and no AMR genes detected. Similarly, *A. salmonicida* from big head carp (Y577) had one plasmid (5,402 bp) and no AMR genes. *A. veronii* from Indian carp (A8-APH) and *A. salmonicida* from chicken (Y47) had three plasmids that had no AMR genes (Supplementary Table S1).

### Transposons detected in the genomes

We found several transposases and integrases in the *Aeromona*s genomes with each isolate having more than six transposases (Table 6). The most dominant transposases were part of insertion sequence (IS) elements; IS481, IS1595, IS110, IS3, IS5 and IS4 that were found in several isolates. Some of the transposases were associated with resistance genes and efflux pumps as shown in Figure 6D that IS5/IS1182 was located close to the tet(E) efflux pump and *tetR* gene in *A. veronii* strain SD21/04. The class I integrase *Int1* was only detected from two fish isolates (SD21-05 and SD21-11).

**Table 6:** Transposases and integrases detected from the *Aeromonas* genomes.

Transposes/Integrases gene description		SD/21-04	SD/21-01	SD/21-05	SD/21-11	XhG1.2	A8-AHP	Phln2	F2S2-1	Y557	Y567	A527	CMF	FC951	VBF557	Y47
Transposes	DDE-type integrase/transposase/recombinase															
	IS5/IS1182 family transposase															
	IS481 family transposase															
	IS1595 family transposase															
	IS110 family transposase															
	IS3 family transposase															
	IS5 family transposase															
	IS66 family transposase															
	IS630 family transposase															
	IS4 family transposase															
	IS21 family transposase															
	IS30-like element ISAs2															
	IS1634 family transposase															
Integrase	site-specific integrase															
	class 1 integron integrase IntI1															
	Integrase															

Blue = presence of genes (detected), white/blank = absence of gene (not detected).

## Discussion

In this study, we have shown that all 15 *Aeromonas* genomes examined had multiple AMR genes suggesting that *Aeromonas* spp. infecting different host species in India could be carriers of multidrug resistance (MDR) genes. We have also shown that WGS is a reliable tool able to profile all AMR genes, efflux pump proteins, integrases, transposes and plasmids present in bacteria genomes, unlike PCR that use primers targeting selected genes posing the danger of missing some of the vital AMR genes encoded in bacteria genomes. In addition, we have shown that pangenome analysis is a reliable tool able to classify members of the genus *Aeromonas* into species by separating the shell genes that are species specific from the core genes shared by all aeromonads. Although the pangenome classified the 15 genomes into four groups based on species, the high similarity of AMR genes determined by phylogenetic analyses is suggestive that there is interspecies transmission of AMR genes among bacteria species isolated from different hosts. This is also indicative that these genes could be part of the conserved genome across the aeromonads being in line with previous observations that Aeromonads are intrinsically resistant to β-lactams (Baron et al., 2017; Kabwe et al., 2020; Sakulworakan et al., 2021).

In general, the ambler classes B, C and D genes accounted for the largest proportion of AMR genes detected from the 15 *Aeromonas* genomes examined. The *Aeromonas* genus contains several aquatic bacteria species both commensals and fish pathogens that host chromosomally located *amp* resistance genes that can be functional (De Luca et al., 2010). Among the class B MBL genes, the high similarity of carbapenem genes *cphA3* and *cphA4* genes from *A. veronii*, *A. hydrophila* and *A. caviae* isolated from humans, Indian carp, sardine, insect, and tilapia demonstrate the ability of different *Aeromonas* sp. isolated from different host species to harbor similar AMR genes. On the other hand, the high similarity of carbapenem gene *cphA5* detected in the *A. salmonicida* genomes from chicken, and butter catfish is suggestive that one *Aeromonas* sp. carrying a similar gene can be a source of AMR transmission to different host species. Wang Y. et al. (2021) and Kabwe et al. (2020) have shown that aeromonads have several MBL genes that include *cphA*, *imiH*, and *ceph-A3* encoded in their chromosomes suggesting that their presence in the *Aeromonas* spp. examined in this study could be that they intrinsically are encoded in the genomes. Despite so, suffice to point out that the MBL genes detected in this study have been reported from different bacteria species isolated from humans, animals, fish, chickens, mussel and the environments in different countries (Maravić et al., 2013; Bottoni et al., 2015; Hilt et al., 2020; Ramsamy et al., 2020; Bertran et al., 2021; Wang Y. et al., 2021). Thus, it is likely that these AMR genes exist in other bacteria species in different aquatic environments and a wide range of host species in India.

As pointed out by Chen et al. (2019) that the diversity of *bla*_OXA_ genes has been expanding to include new variants of *bla*_OXA-12_ such as *bla*_OXA-427_, *bla*_OXA-724_ and *bla*_OXA-780_, all detected in this study. We found *bla*_OXA-12_ in sequences of four *A. veronii* isolates obtained from humans, catfish, carp, and insect. Previously, it was detected in *Aeromonas* spp. such as *A. hydrophila*, *A. allosaccharophila*, *A. veronii*, and *A. rivipollensis* isolated from humans, chicken, pork, and wild nutria (*Myocastor coypus*) (Park et al., 2018; Shen et al., 2018; Xiao et al., 2020), respectively. We also found *bla*_OXA-427_ in *A. salmonicida* isolated from bighead fish, butter catfish and prawn, which is an emerging class D carbapenemase that confer resistance against a wide range of β-lactams including broad-spectrum penicillins, cephalosporins, and carbapenems (Bogaerts et al., 2015, 2017). Although outbreaks in humans have only been reported from hospitals in Belgium where *bla*_OXA-427_ was isolated from nosocomial *Klebsiella pneumoniae* and *Enterobacter cloacae* infections (Desmet et al., 2018), its presence among *Aeromonas* spp. shows global distribution involving humans, animals and fish. For example, it has been detected from *A. caviae* and *A. hydrophila* isolated from humans in China (Tang et al., 2020; Lin et al., 2021), *Aeromonas* spp. from reservoir water in Singapore (Zhong et al., 2021), *A. media* in Nebraska watershed (Donner et al., 2022), *A. salmonicida* from Atlantic salmon (*Salmo salar* L) in Chile (Vásquez-Ponce et al., 2022), and pork processing plant in Spain (Cobo-Díaz et al., 2021). Similarly, bla_OXA-724_ has been detected from *A. dhakensis* isolated from humans in Spain (Bertran et al., 2021), *A. hydrophila* from pigs in South Africa (Ramsamy et al., 2021) as well as *A. jandaei* and *A. hydrophila* from chicken and catfish in the United States (Wang Y. et al., 2021), while in the present study it was found in *A. hydrophila* from carp and *A. veronii* from sardine from India. *bla*_OXA-72_ has been detected from *Acinetobacter baumannii* in humans where it has been associated with pneumonia, septic shock, and respiratory failure (Jia et al., 2019). Given that various bla_OXA_ genes have been shown to be intrinsically encoded in the chromosomes of various *Aeromonas* spp. (Kabwe et al., 2020; Wang Y. et al., 2021), these findings show that *Aeromonas* spp. found in different host species and aquatic environment could play a vital role in the global spread of emerging β-lactam resistance genes such as *bla*_OXA-427_ and *bla*_OXA-724_.

In this study, we detected class C β-lactamase genes from 10 of the 15 *Aeromonas* genomes examined unlike class D genes that were detected in all genomes. As shown in our findings, class C genes comprised of the cephalosporin/penam *cepS*, *bla*_MOX_ and *bla*_FOX_ genes as well as the *bla*_AQU-2_ β-lactamase gene. Among these, *cepS* has previously been detected from various *Aeromonas* spp. isolated from humans, pigs, catfish, chicken, frogs, mullet and the environment while *bla*_AQU-2_ has been reported from *Aeromonas* spp. isolated from humans and chicken in different countries (Walsh et al., 1997; Ramadan et al., 2018; Seo and Lee, 2018; Wang et al., 2019; Bertran et al., 2021; Kimera et al., 2021; Wang Y. et al., 2021). Similarly, *bla*_FOX-2_, *bla*_FOX- 4_, *bla*_FOX- 5_ and *bla*_MOX-7_, have been detected from different bacteria species including *Aeromonas* spp. isolated from humans, wastewater, fish tanks, mussel, and wild animals in different countries (Maravić et al., 2013; Bertran et al., 2021). Altogether, these studies show that the class C β-lactamase genes are prevalent in a wide range of host species in various countries indicating they could be present in several other species not included in this study found in India. Suffice to point out that *cepS*, *bla*_AQU-2_, and *bla*_FOX/MOX_ have been detected in the chromosomes of various *Aeromonas* spp. (Kabwe et al., 2020; Wang Y. et al., 2021), suggesting that the class C β-lactamase genes detected in this study could have been intrinsically encoded in the genomes of the *Aeromonas* spp. examined. This is also supported by the high prevalence of the *crp* gene detected in nine of the 15 genomes examined in this study, which is a RND efflux pump associated with resistance against penam, cephalosporin, macrolide, trimethoprim and fluoroquinolone (Nishino et al., 2008). This finding points to its wide prevalence among *Aeromonas* spp. infecting humans, fish, insect and animals in India. Previously, *crp* has been found in *Cronobacter* spp. isolated from infant food (Carvalho et al., 2020), *C. sakazakii* from powdered milk (Holý et al., 2020), *Enterobacter hormaechi* from yoghurt (Tóth et al., 2020), *Salmonella enterica* from ducks, (Yu et al., 2022), and *Vibrio* spp. from human and environmental samples (Pérez-Duque et al., 2021; Nguyen et al., 2022).

Several studies have shown that environmental aeromonads contain chromosomally encoded β-lactamases that cause resistance to drugs including ampicillins, cephalosporin and penicillin (Richardson et al., 1982; Zemelman et al., 1984; Motyl et al., 1985; Shannon et al., 1986; Chang and Bolton, 1987; Fosse et al., 2003; Girlich et al., 2011). Thus, it is likely that the resistance observed against ampicillin, penicillin and cephalosporin in our phenotypic analysis was encoded in genomes of *Aeromonas* spp. examined. So, it can be speculated that Aeromonads could be an important source for the spread of novel β-lactamases to human clinically important bacteria in line with Fosse et al. (2003) and Girlich et al. (2011), who pointed out that the resistance originating from aeromonads poses a significant public health risk to humans.

The resistance against gentamycin in the genus *Aeromonas* has been linked to variable results as shown that gentamycin sensitive *Aeromonas* spp. have previously been isolated from rainbow trout (*Oncorhynchus mykiss*) (Akinbowale et al., 2007), carp (Öztürk et al., 2007) and Nile crocodile (*Crocodylus niloticus*) (Turutoglu et al., 2005) while gentamycin resistant *Aeromonas* spp. have been isolated from catfish (Chinedu et al., 2020) and European rivers (Goñi-Urriza et al., 2000). Hence, it is unknown whether the *aadA2* and *ANT(3″)-IIa* aminoglycoside resistance observed in our fish isolates was intrinsically or extrinsically acquired. Detection of the *ANT(3″)-IIa* aminoglycoside gene linked to *intl1* the integrase together with the chloramphenicol *cmlAI* and MFS *QacEdelta-1* efflux pumps in *A. caviae* strain SD/21–11 in this study is suggestive that there might be some transfer or acquisition of gentamycin genes into *Aeromonas* genomes. This is supported with observations seen in *A. hydrophila* strain SD/21–05 that also had the *intl* integrase linked to the aminoglycoside (*aadA2*), sulfonamide (*sul1*) and trimethoprim (*dfr12*) genes together with the MFS *QacEdelta-1* efflux pump pointing to transfer or acquisition of chloramphenicol, trimethoprim and gentamycin resistance genes into *Aeromonas* genomes. Even though several studies (Koksal et al., 2007; Awan et al., 2009; Saengsitthisak et al., 2020; Dhanapala et al., 2021) have reported erythromycin resistance in *Aeromonas* spp. suggesting that it could be chromosomally integrated, isolates of *A. sobria* from prawn (*Penaus monodon*) (Vaseeharan et al., 2005), *A. veronii* from sea bass (*Lateolabrax maculatus*) (Wang B. et al., 2021) and *A. hydrophila* from humans (Von Graevenitz and Mensch, 1968) were shown to be sensitive to erythromycin. Although our fish isolates showed resistance to erythromycin, it is unknown whether the resistance was intrinsic or extrinsically acquired. However, it is likely that the resistance seen against tetracycline, sulfonamide and trimethoprim could have been acquired from treatment of diseased fish using these antibiotics as reported from clinical reports. Moreover, these antibiotics are widely used in aquaculture in India and resistance based on disc diffusion test has been reported previously (Abraham et al., 2017; Roy et al., 2021; Sivaraman et al., 2021; Patil et al., 2022).

Our findings show that all 15 *Aeromonas* genomes had multidrug efflux pump proteins. The *mdtL* protein which is one of the first line of defence against antimicrobials involved in decreasing intracellular drugs levels (Rahman et al., 2017) was detected in most isolates. *mdtL* has been shown to increase resistance against fosfomycin and chloramphenicol (Kvist et al., 2008). Among the major facilitator superfamily (MFS), *emrB* and *emrD* involved in resistance against several drugs like norfloxacin, tetracycline, chloramphenicol, novobiocin, fluoroquinolone and nalidixic acid (Jahan et al., 2021) were detected in several isolates. As for the RND proteins, we detected the *tet(E)* gene known to encode the tetracycline efflux pumps (Møller et al., 2016). Other multidrug efflux pump proteins detected include *rarD*, *qnr*, *mdtH*, *mdtD, pbp1A* and *qacEdelta1* involved in resistance against chloramphenicol, fluoroquinolone, novobiocin amoxicillin, and several other drugs (Kazama et al., 1999; Nagakubo et al., 2002; Stanhope et al., 2008; Ovchinnikov et al., 2015; Zago et al., 2020). In the present study, the MFS *QacEdelta-1* efflux pump gene was linked to the trimethoprim (*dfrA12*), aminoglycoside (*aad2*) and sulfonamide (*sul2*) resistance genes in *A. hydrophila* strain SD/21–05 while the tet(E) pump was linked to the *tetR* tetracycline gene in *A. veronii* strain SD/21–04. In *A. caviae* strain SD/21–11, the chloramphenicol *cmlA1* and MFS *QacEdelta-1* efflux pumps gene were linked to the *ANT(3″)-IIa* aminoglycoside gene and *sul1* sulfonamide genes whereas in *A. hydrophila* strain SD/21–01 the multidrug complex OprM-MexB and RND smeD efflux pumps were linked to the tetracycline *tetR* gene. We also detected *fosC2*, *blrB*, *BRP*, and *BMC* involved in resistance against fosfomycin, β-lactams, glycopeptide, and bicyclomycin (Galm et al., 2005; Nikolaidis et al., 2014; Jahan et al., 2021). These findings concur with previous studies (Li and Nikaido, 2004, 2009) showing that AMR genes expressed by *Aeromonas* spp. are often linked to multidrug resistance proteins.

The most important mobile genetic elements (MGEs) known to play a key role in the spread of AMR genes include class 1 integrons (*intI*), transposons and plasmids (Liebert et al., 1999; Carattoli, 2001; Stalder et al., 2012). In this study, *intI* was only detected from two out of the four isolates sequenced in this study. In *A. hydrophila* strain SD/21–05, it was located next to the trimethoprim *dfrA12*, aminoglycoside *aadA2* and sulphonamide *sul1* genes, whereas in *A. caviae* strain SD/21–11 it was linked to aminoglycoside *ANT(3″)-IIa*, chloramphenicol *cmlA1* and sulphonamide *sul1* genes. These findings are in line with Ranjbar et al. (2019), Pérez-Valdespino et al. (2009), and Schmidt et al. (2001) who found *sul1, dfrA12*, *aadA2*, *aadA1*, *bla_OXA_, cmlA4* and *ANT(3″)* gene cassettes in sequences linked to *intI* obtained from different *Aeromonas* spp. The IS classes of transposases reported in this study corroborates with several studies that found IS66, IS30, IS3, IS4, IS5, IS66, IS630, ISA110, ISA1182 transposases in *Aeromonas* spp. isolated from aquatic environments and different host species (Najimi et al., 2009; Studer et al., 2013; Adamczuk and Dziewit, 2017; Vincent et al., 2017; Jin et al., 2020; Ragupathi et al., 2020). In the present study, some transposases were linked to multidrug efflux pumps and AMR genes as shown that the IS5/IS1182 transposase was linked to the Tet(E) efflux pump and tetracycline *tetR* gene in *A. veronii* strain SD/21–04. These transposases have also been found in plasmids linked to AMR (Najimi et al., 2009). For example, IS630 and IS600 were found in the pFBAOT6 plasmid linked to tetracycline resistance in *A. caviae* (Rhodes et al., 2004) while IS4 was found in the Inc-Q3 plasmid showing resistance against quinoline in *Aeromonas* spp. (Piotrowska et al., 2020). Finally, the detection of genes like *D-met*, *mbeD*, *mobDI*, *parB*, *repB*, and *relE* (Carattoli, 2001) in the plasmids of *Aeromonas* spp. shows that the identified plasmids in this study had the potential to transfer AMR genes to other bacteria.

## Conclusion

In this study, we have shown that *Aeromonas* spp. isolated from fish, prawn, insect, chicken and humans in India carry various AMR genes. The sequenced isolates of aeromonads from aquaculture reveal well-known AMR genes and class 1 integrons documented from similar studies from aquaculture worldwide, while aeromonads from other environmental sources do not contain commonly transferable AMR genes. These findings also showed high similarity of AMR genes found in different *Aeromonas* spp. despite the bacteria being isolated from different host species. Thus, we advocate that the control of AMR caused by *Aeromonas* spp. in India should be done using a One Health approach.

## Data availability statement

The data presented in the study are deposited in the NCBI repository, available at: https://www.ncbi.nlm.nih.gov/nuccore/JAJVCT000000000, https://www.ncbi.nlm.nih.gov/nuccore/JAJVCU000000000, https://www.ncbi.nlm.nih.gov/nuccore/JAJVCV000000000, and https://www.ncbi.nlm.nih.gov/nuccore/JAJVCW000000000.1.

## Author contributions

SD, HS, and HM: conceptualization, methodology, data curation, formal analysis, manuscript preparation, and resources. EA-W, JK, BP, InK, IdK, and ØE: data analysis and preparation of manuscript. All authors contributed to the article and approved the submitted version.

## Funding

This study was financed by the Research Council of Norway (FIFOSA-21 Project) Grant Number 320692. The study was also funded by the National Natural Science Foundation of China (Nos. 31872602, 32061133007, 31822058).

## Conflict of interest

The authors declare that the research was conducted in the absence of any commercial or financial relationships that could be construed as a potential conflict of interest.

## Publisher’s note

All claims expressed in this article are solely those of the authors and do not necessarily represent those of their affiliated organizations, or those of the publisher, the editors and the reviewers. Any product that may be evaluated in this article, or claim that may be made by its manufacturer, is not guaranteed or endorsed by the publisher.

## References

[ref1] Abdelsalam M. Ewiss M. Z. Khalefa H. S. Mahmoud M. A. Elgendy M. Y. Abdel-Moneam D. A. (2021). Coinfections of Aeromonas spp., enterococcus faecalis, and vibrio alginolyticus isolated from farmed Nile tilapia and African catfish in Egypt, with an emphasis on poor water quality. Microb. Pathog. 160:105213. doi: 10.1016/j.micpath.2021.105213 34582943

[ref2] Abraham T. J. Anwesha R. Julinta R. B. Singha J. Patil P. K. (2017). Efficacy of oxytetracycline and potentiated sulphonamide oral therapies against Aeromonas hydrophila infection in Nile tilapia Oreochromis niloticus. J. Coast. Life Med. 5, 371–374. doi: 10.12980/jclm.5.2017j7-89

[ref3] Adamczuk M. Dziewit L. (2017). Genome-based insights into the resistome and mobilome of multidrug-resistant Aeromonas sp. ARM81 isolated from wastewater. Arch. Microbiol. 199:7. doi: 10.1007/s00203-016-1285-6 PMC5216076 27590015

[ref4] Akinbowale O. L. Peng H. Grant P. Barton M. D. (2007). Antibiotic and heavy metal resistance in motile aeromonads and pseudomonads from rainbow trout (Oncorhynchus mykiss) farms in Australia. Int. J. Antimicrob. Agents 30, 177–182. doi: 10.1016/j.ijantimicag.2007.03.012, PMID: 17524624

[ref5] Alcock B. P. Raphenya A. R. Lau T. T. Tsang K. K. Bouchard M. Edalatmand A.. (2020). CARD 2020: antibiotic resistome surveillance with the comprehensive antibiotic resistance database. Nucleic Acids Res. 48, D517–D525. doi: 10.1093/nar/gkz935, PMID: 31665441 PMC7145624

[ref6] Awan M. B. Maqbool A. Bari A. Krovacek K. (2009). Antibiotic susceptibility profile of Aeromonas spp. isolates from food in Abu Dhabi, United Arab Emirates. New Microbiol. 32, 17–23. 19382665

[ref7] Bankevich A. Nurk S. Antipov D. Gurevich A. A. Dvorkin M. Kulikov A. S.. (2012). SPAdes: a new genome assembly algorithm and its applications to single-cell sequencing. J. Comput. Biol. 19, 455–477. doi: 10.1089/cmb.2012.0021, PMID: 22506599 PMC3342519

[ref8] Baron S. Granier S. A. Larvor E. Jouy E. Cineux M. Wilhelm A.. (2017). Aeromonas diversity and antimicrobial susceptibility in freshwater—an attempt to set generic epidemiological cut-off values. Front. Microbiol. 8:503. doi: 10.3389/fmicb.2017.00503 28400760 PMC5368242

[ref9] Becker L. Steglich M. Fuchs S. Werner G. Nübel U. (2016). Comparison of six commercial kits to extract bacterial chromosome and plasmid DNA for MiSeq sequencing. Sci. Rep. 6, 1–5. doi: 10.1038/srep28063 27312200 PMC4911584

[ref10] Bertran X. Rubio M. Gómez L. Llovet T. Muñoz C. Navarro F.. (2021). Taxonomic identification of different species of the genus Aeromonas by whole-genome sequencing and use of their species-specific β-lactamases as phylogenetic markers. Antibiotics 10:354. doi: 10.3390/antibiotics10040354, PMID: 33800590 PMC8065696

[ref11] Bioinformatics B. (2011). FastQC: A Quality Control Tool for High Throughput Sequence data. Cambridge, UK: Babraham Institute.

[ref12] Bogaerts P. Naas T. Saegeman V. Bonnin R. A. Schuermans A. Evrard S.. (2017). OXA-427, a new plasmid-borne carbapenem-hydrolysing class D β-lactamase in Enterobacteriaceae. J. Antimicrob. Chemother. 72, 2469–2477. doi: 10.1093/jac/dkx184, PMID: 28859446

[ref13] Bogaerts P. Thierry N. Evrard S. Bouchahrouf W. Saegeman V. Lasserre C.. (2015). OXA-427, a new plasmidic ESBL class D OXA-carbapenemase recovered from Enterobacteriaceae clinical isolates Abstr ECCMID 2015, abstr, P1307.

[ref14] Bolger A. M. Lohse M. Usadel B. (2014). Trimmomatic: a flexible trimmer for Illumina sequence data. Bioinformatics 30, 2114–2120. doi: 10.1093/bioinformatics/btu170, PMID: 24695404 PMC4103590

[ref15] Bottoni C. Marcoccia F. Compagnoni C. Colapietro M. Sabatini A. Celenza G.. (2015). Identification of new natural CphA metallo-β-lactamases CphA4 and CphA5 in Aeromonas veronii and Aeromonas hydrophila isolates from municipal sewage in Central Italy. Antimicrob. Agents Chemother. 59, 4990–4993. doi: 10.1128/AAC.00628-15, PMID: 25987617 PMC4505251

[ref16] Carattoli A. (2001). Importance of integrons in the diffusion of resistance. Vet. Res. 32, 243–259. doi: 10.1051/vetres:2001122, PMID: 11432416

[ref17] Carvalho G. G. Calarga A. P. Teodoro J. R. Queiroz M. M. Astudillo-Trujillo C. A. Levy C. E.. (2020). Isolation, comparison of identification methods and antibiotic resistance of Cronobacter spp. in infant foods. Food Res. Int. 137:109643. doi: 10.1016/j.foodres.2020.109643, PMID: 33233222

[ref18] Chang B. J. Bolton S. M. (1987). Plasmids and resistance to antimicrobial agents in Aeromonas sobria and Aeromonas hydrophila clinical isolates. Antimicrob. Agents Chemother. 31, 1281–1282. doi: 10.1128/AAC.31.8.1281, PMID: 3631947 PMC174920

[ref19] Chen Q. Zhou W. Qian C. Shen K. Zhu X. Zhou D.. (2019). OXA-830, a novel chromosomally encoded extended-spectrum class D β-lactamase in Aeromonas simiae. Front. Microbiol. 10:2732. doi: 10.3389/fmicb.2019.02732, PMID: 31849884 PMC6902050

[ref20] Chinedu O. Iniobong A. D. Chidinma W.-E. (2020). Report on multiple antibiotics resistance Aeromonas hydrophila isolated from catfish farms in Epe Lagos. Middle East J. Appl. Sci. Technol. 3, 51–57.

[ref21] Cobo-Díaz J. F. Alvarez-Molina A. Alexa E. A. Walsh C. J. Mencía-Ares O. Puente-Gómez P.. (2021). Microbial colonization and resistome dynamics in food processing environments of a newly opened pork cutting industry during 1.5 years of activity. Microbiome 9, 1–19. doi: 10.1186/s40168-021-01131-9 34645520 PMC8515711

[ref22] Cockerill F. R. Wikler M. Bush K. Dudley M. Eliopoulos G. Hardy D.. (2012). Performance Standards for Antimicrobial Susceptibility Testing: Twenty-second Informational Supplement. Wayne: Clinical and Laboratory Standards Institute.

[ref23] Cohen J. (1968). Weighted kappa: nominal scale agreement provision for scaled disagreement or partial credit. Psychol. Bull. 70, 213–220. doi: 10.1037/h0026256 19673146

[ref24] Coil D. Jospin G. Darling A. E. (2015). A5-miseq: an updated pipeline to assemble microbial genomes from Illumina MiSeq data. Bioinformatics 31, 587–589. doi: 10.1093/bioinformatics/btu661, PMID: 25338718

[ref25] Das S. Aswani R. Jasim B. Sebastian K. Radhakrishnan E. Mathew J. (2020). Distribution of multi-virulence factors among Aeromonas spp. isolated from diseased Xiphophorus hellerii. Aquac. Int. 28, 235–248. doi: 10.1007/s10499-019-00456-5

[ref26] Das S. Sreejith S. Babu J. Francis C. Midhun J. Aswani R.. (2021). Genome sequencing and annotation of multi-virulent Aeromonas veronii XhG1. 2 isolated from diseased Xiphophorus hellerii. Genomics 113, 991–998. doi: 10.1016/j.ygeno.2020.10.034, PMID: 33144215

[ref27] De Luca F. Giraud-Morin C. Rossolini G. M. Docquier J.-D. Fosse T. (2010). Genetic and biochemical characterization of TRU-1, the endogenous class C β-lactamase from Aeromonas enteropelogenes. Antimicrob. Agents Chemother. 54, 1547–1554. doi: 10.1128/AAC.01252-09, PMID: 20124004 PMC2849387

[ref28] Desmet S. Nepal S. van Dijl J. M. Van Ranst M. Chlebowicz M. A. Rossen J. W.. (2018). Antibiotic resistance plasmids cointegrated into a megaplasmid harboring the Bla OXA-427 carbapenemase gene. Antimicrob. Agents Chemother. 62, e01448–e01417. doi: 10.1128/AAC.01448-17 29311088 PMC5826099

[ref29] Dhanapala P. M. Kalupahana R. S. Kalupahana A. W. Wijesekera D. Kottawatta S. A. Jayasekera N. K.. (2021). Characterization and antimicrobial resistance of environmental and clinical Aeromonas species isolated from fresh water ornamental fish and associated farming environment in Sri Lanka. Microorganisms 9:2106. doi: 10.3390/microorganisms9102106, PMID: 34683427 PMC8537582

[ref30] Donner L. Staley Z. R. Petali J. Sangster J. Li X. Mathews W.. (2022). The human health implications of antibiotic resistance in environmental isolates from two Nebraska watersheds. Microbiol. Spectr. 10, e02082–e02021. doi: 10.1128/spectrum.02082-21 35311538 PMC9045274

[ref31] Dubey S. Maiti B. Girisha S. K. Das R. Lamkhannat M. Mutoloki S.. (2021). Aeromonas species obtained from different farmed aquatic species in India and Taiwan show high phenotypic relatedness despite species diversity. BMC. Res. Notes 14, 1–8. doi: 10.1186/s13104-021-05716-3 34399833 PMC8365956

[ref32] Elgendy M. Y. Soliman W. S. Abbas W. T. Ibrahim T. B. Younes A. M. Omara S. T. (2017). Investigation of some virulence determents in Aeromonas hydrophila strains obtained from different polluted aquatic environments. Jordan J. Biol. Sci. 10, 265–272.

[ref33] Figueras M. J. Beaz-Hidalgo R. (2015). Aeromonas infections in humans. Aeromonas, ed. Graf J. (Norfolk, UK: Caister Academic Press). 65–108.

[ref34] Fosse T. Giraud-Morin C. Madinier I. (2003). Phénotypes de résistance aux β-lactamines dans le genre Aeromonas. Pathol. Biol. 51, 290–296. doi: 10.1016/S0369-8114(03)00027-0, PMID: 14567197

[ref35] Gaio D. Anantanawat K. To J. Liu M. Monahan L. Darling A. E. (2021). Hackflex: low cost Illumina Nextera flex sequencing library construction. BioRxiv 779215. doi: 10.1099/mgen.0.000744 PMC8914357 35014949

[ref36] Galm U. Hager M. H. Van Lanen S. G. Ju J. Thorson J. S. Shen B. (2005). Antitumor antibiotics: bleomycin, enediynes, and mitomycin. Chem. Rev. 105, 739–758. doi: 10.1021/cr030117g, PMID: 15700963

[ref37] Girlich D. Poirel L. Nordmann P. (2011). Diversity of clavulanic acid-inhibited extended-spectrum β-lactamases in Aeromonas spp. from the Seine River, Paris, France. Antimicrob. Agents Chemother. 55, 1256–1261. doi: 10.1128/AAC.00921-10, PMID: 21149627 PMC3067085

[ref38] Gogry F. A. Siddiqui M. T. (2019). Emergence of mcr-1 conferred colistin resistance among bacterial isolates from urban sewage water in India. Environ. Sci. Pollut. Res. 26, 33715–33717. doi: 10.1007/s11356-019-06561-5, PMID: 31625114

[ref39] Goñi-Urriza M. Pineau L. Capdepuy M. Roques C. Caumette P. Quentin C. (2000). Antimicrobial resistance of mesophilic Aeromonas spp. isolated from two European rivers. J. Antimicrob. Chemother. 46, 297–301. doi: 10.1093/jac/46.2.297, PMID: 10933657

[ref40] Guan G. He X. Chen J. Bin L. Tang X. (2020). Identifying the mechanisms underlying the protective effect of tetramethylpyrazine against cisplatin-induced in vitro ototoxicity in HEI-OC1 auditory cells using gene expression profiling. Mol. Med. Rep. 22, 5053–5068. doi: 10.3892/mmr.2020.11631, PMID: 33174043 PMC7646960

[ref41] Hadfield J. Croucher N. J. Goater R. J. Abudahab K. Aanensen D. M. Harris S. R. (2018). Phandango: an interactive viewer for bacterial population genomics. Bioinformatics 34, 292–293. doi: 10.1093/bioinformatics/btx610, PMID: 29028899 PMC5860215

[ref42] Harikrishnan R. Balasundaram C. (2005). Modern trends in Aeromonas hydrophila disease management with fish. Rev. Fish. Sci. 13, 281–320. doi: 10.1080/10641260500320845

[ref43] Hilt E. E. Fitzwater S. P. Ward K. de St Maurice A. Chandrasekaran S. Garner O. B.. (2020). Carbapenem resistant Aeromonas hydrophila carrying blacphA7 isolated from two solid organ transplant patients. Frontiers in cellular and infection. Microbiology 624, 563350–563482. doi: 10.3389/fcimb.2020.563482 PMC7649429 33194801

[ref44] Holý O. Parra-Flores J. Lepuschitz S. Alarcón-Lavín M. P. Cruz-Córdova A. Xicohtencatl-Cortes J.. (2020). Molecular characterization of cronobacter sakazakii strains isolated from powdered milk. Foods 10:20. doi: 10.3390/foods10010020, PMID: 33374633 PMC7822459

[ref45] Jahan M. I. Rahaman M. M. Hossain M. A. Sultana M. (2021). Draft genome sequence of a carbapenem-resistant clinical Acinetobacter baumannii revealing co-existence of four classes of β-lactamases. J. Glob. Antimicrob. Resist. 27, 329–331. doi: 10.1016/j.jgar.2021.11.002, PMID: 34800708

[ref46] Janda J. M. Abbott S. L. (2010). The genus Aeromonas: taxonomy, pathogenicity, and infection. Clin. Microbiol. Rev. 23, 35–73. doi: 10.1128/CMR.00039-09, PMID: 20065325 PMC2806660

[ref47] Jayasankar P. (2018). Present status of freshwater aquaculture in India-a review. Indian J. Fish. 65, 157–165. doi: 10.21077/ijf.2018.65.4.81300-20

[ref48] Jazayeri H. Raz A. Favia G. Ricci I. Zakeri S. (2011). Identification of the midgut microbiota of an. Stephensi and an. Maculipennis for their application as a paratransgenic tool against malaria. PLoS One 6:e28484. doi: 10.1371/journal.pone.0028484 22163022 PMC3232223

[ref50] Jia H. Sun Q. Ruan Z. Xie X. (2019). Characterization of a small plasmid carrying the carbapenem resistance gene blaOXA-72 from community-acquired Acinetobacter baumannii sequence type 880 in China. Infect. Drug Resist. 12, 1545–1553. doi: 10.2147/IDR.S202803, PMID: 31239730 PMC6559137

[ref51] Jin L. Chen Y. Yang W. Qiao Z. Zhang X. (2020). Complete genome sequence of fish-pathogenic Aeromonas hydrophila HX-3 and a comparative analysis: insights into virulence factors and quorum sensing. Sci. Rep. 10, 1–15. doi: 10.1038/s41598-020-72484-8 32968153 PMC7512022

[ref52] Jones D. T. Taylor W. R. Thornton J. M. (1992). The rapid generation of mutation data matrices from protein sequences. Bioinformatics 8, 275–282. doi: 10.1093/bioinformatics/8.3.275, PMID: 1633570

[ref53] Joseph N. M. Sistla S. Dutta T. K. Badhe A. S. Rasitha D. Parija S. C. (2011). Reliability of Kirby-Bauer disk diffusion method for detecting meropenem resistance among non-fermenting gram-negative bacilli. Indian J. Pathol. Microbiol. 54, 556–560. doi: 10.4103/0377-4929.85092, PMID: 21934220

[ref54] Joshi H. (2016). Isolation, identification, and antibiotics resistance of Aeromonas spp. from lakes of Udaipur (Rajasthan). India. Asian J. Pharm. 10, 132–136. doi: 10.22377/ajp.v10i2.612

[ref55] Kabwe M. Brown T. Speirs L. Ku H. Leach M. Chan H. T.. (2020). Novel bacteriophages capable of disrupting biofilms from clinical strains of Aeromonas hydrophila. Front. Microbiol. 11:194. doi: 10.3389/fmicb.2020.00194, PMID: 32117183 PMC7033617

[ref56] Kahlmeter G. Brown D. Goldstein F. MacGowan A. Mouton J. Odenholt I.. (2006). European committee on antimicrobial susceptibility testing (EUCAST) technical notes on antimicrobial susceptibility testing. Wiley Online Libr. 12, 501–503. doi: 10.1111/j.1469-0691.2006.01454.x 16700696

[ref57] Kaskhedikar M. Chhabra D. (2010). Multiple drug resistance in Aeromonas hydrophila isolates of fish. Food Microbiol. 28, 157–168.

[ref58] Kaspersen H. Fiskebeck E. Z. Sekse C. Slettemeås J. S. Urdahl A. M. Norström M.. (2020). Comparative genome analyses of wild type-and quinolone resistant Escherichia coli indicate dissemination of QREC in the Norwegian broiler breeding pyramid. Front. Microbiol. 11:938. doi: 10.3389/fmicb.2020.00938, PMID: 32508776 PMC7248565

[ref59] Kazama H. Hamashima H. Sasatsu M. Arai T. (1999). Characterization of the antiseptic-resistance gene qacE Δ 1 isolated from clinical and environmental isolates of Vibrio parahaemolyticus and vibrio cholerae non-O1. FEMS Microbiol. Lett. 174, 379–384. doi: 10.1111/j.1574-6968.1999.tb13593.x 10339831

[ref60] Kimera Z. I. Mgaya F. X. Misinzo G. Mshana S. E. Moremi N. Matee M. I. (2021). Multidrug-resistant, including extended-spectrum beta lactamase-producing and quinolone-resistant, Escherichia coli isolated from poultry and domestic pigs in Dar Es Salaam. Tanzania. Antib. 10:406. doi: 10.3390/antibiotics10040406, PMID: 33918543 PMC8069735

[ref61] Koksal F. Oguzkurt N. Samastı M. Altas K. (2007). Prevalence and antimicrobial resistance patterns of Aeromonas strains isolated from drinking water samples in Istanbul. Turkey. Chemother. 53, 30–35. doi: 10.1159/000098248, PMID: 17191011

[ref62] Kumar S. Stecher G. Tamura K. (2016). MEGA7: molecular evolutionary genetics analysis version 7.0 for bigger datasets. Mol. Biol. Evol. 33, 1870–1874. doi: 10.1093/molbev/msw054, PMID: 27004904 PMC8210823

[ref63] Kuncham R. Sivaprakasam T. Kumar R. P. Sreenath P. Nayak R. Thayumanavan T.. (2017). Bacterial fauna associating with chironomid larvae from lakes of Bengaluru city, India-a 16s rRNA gene based identification. Genom. Data 12, 44–48. doi: 10.1016/j.gdata.2017.03.001, PMID: 28316932 PMC5342978

[ref64] Kvist M. Hancock V. Klemm P. (2008). Inactivation of efflux pumps abolishes bacterial biofilm formation. Appl. Environ. Microbiol. 74, 7376–7382. doi: 10.1128/AEM.01310-08, PMID: 18836028 PMC2592912

[ref65] Li X.-Z. Nikaido H. (2004). Efflux-mediated drug resistance in bacteria. Drugs 64, 159–204. doi: 10.2165/00003495-200464020-00004 14717618

[ref66] Li X.-Z. Nikaido H. (2009). Efflux-mediated drug resistance in bacteria. Drugs 69, 1555–1623. doi: 10.2165/11317030-000000000-00000, PMID: 19678712 PMC2847397

[ref67] Liebert C. A. Hall R. M. Summers A. O. (1999). Transposon Tn 21, flagship of the floating genome. Microbiol. Mol. Biol. Rev. 63, 507–522. doi: 10.1128/MMBR.63.3.507-522.1999, PMID: 10477306 PMC103744

[ref68] Lijon M. B. Khatun M. M. Islam A. Khatun M. M. Islam M. A. (2015). Detection of multidrug resistance Aeromonas hydrophila in farm raised fresh water prawns. J. Adv.Vet. Anim. Res. 2, 469–474. doi: 10.5455/javar.2015.b120

[ref69] Lin X. Lu J. Qian C. Lin H. Li Q. Zhang X.. (2021). Molecular and functional characterization of a novel plasmid-borne blaNDM-like gene, blaAFM-1, in a clinical strain of *Aeromonas hydrophila*. Infect. Drug Resist. 14, 1613–1622. doi: 10.2147/IDR.S297419, PMID: 33911885 PMC8075316

[ref70] Lulijwa R. Rupia E. J. Alfaro A. C. (2020). Antibiotic use in aquaculture, policies and regulation, health and environmental risks: a review of the top 15 major producers. Rev. Aquac. 12, 640–663. doi: 10.1111/raq.12344

[ref71] Maravić A. Skočibušić M. Šamanić I. Fredotović Ž. Cvjetan S. Jutronić M.. (2013). Aeromonas spp. simultaneously harbouring blaCTX-M-15, blaSHV-12, blaPER-1 and blaFOX-2, in wild-growing Mediterranean mussel (Mytilus galloprovincialis) from Adriatic Sea, Croatia. Int. J. Food Microbiol. 166, 301–308. doi: 10.1016/j.ijfoodmicro.2013.07.010, PMID: 23973842

[ref72] Misra S. K. Shimada T. Bhadra R. K. Pal S. C. Nair G. B. (1989). Serogroups of Aeromonas species from clinical and environmental sources in Calcutta. India. J. Diarrh. Dis. Res. 7, 8–12. 2607101

[ref73] Møller T. S. Overgaard M. Nielsen S. S. Bortolaia V. Sommer M. O. Guardabassi L.. (2016). Relation between tetR and tetA expression in tetracycline resistant Escherichia coli. BMC Microbiol. 16, 1–8. doi: 10.1186/s12866-016-0649-z 26969122 PMC4788846

[ref74] Motukupally S. R. Singh A. Garg P. Sharma S. (2014). Microbial keratitis due to aeromonas species at a tertiary eye care center in southern India. Asia-Pacific J. Ophthalmol. 3, 294–298. doi: 10.1097/APO.0000000000000018, PMID: 26107916

[ref75] Motyl M. R. McKinley G. Janda J. M. (1985). In vitro susceptibilities of Aeromonas hydrophila, Aeromonas sobria, and Aeromonas caviae to 22 antimicrobial agents. Antimicrob. Agents Chemother. 28, 151–153. doi: 10.1128/AAC.28.1.151, PMID: 4037775 PMC176330

[ref76] Nadiga M. Vaidyanathan V. Thayumanavan T. (2016). Draft genome sequence of Aeromonas dhakensis strain F2S2-1, isolated from the skin surface of an Indian oil sardine (Sardinella longiceps). Genome Announc. 4, e00494–e00416. doi: 10.1128/genomeA.00494-16 27540048 PMC4991693

[ref77] Nagakubo S. Nishino K. Hirata T. Yamaguchi A. (2002). The putative response regulator BaeR stimulates multidrug resistance of Escherichia coli via a novel multidrug exporter system. MdtABC. J. Bacteriol. 184, 4161–4167. doi: 10.1128/JB.184.15.4161-4167.2002, PMID: 12107133 PMC135206

[ref78] Nagar V. Shashidhar R. Bandekar J. R. (2011). Prevalence, characterization, and antimicrobial resistance of Aeromonas strains from various retail food products in Mumbai. India. J. Food Sci. 76, M486–M492. doi: 10.1111/j.1750-3841.2011.02303.x, PMID: 21824136

[ref79] Najimi M. Balado M. Lemos M. L. Osorio C. R. (2009). Genetic characterization of pAsa6, a new plasmid from Aeromonas salmonicida subsp. salmonicida that encodes a type III effector protein AopH homolog. Plasmid 61, 176–181. doi: 10.1016/j.plasmid.2009.01.001, PMID: 19195484

[ref80] Nayduch D. Pittman Noblet G. Stutzenberger F. J. (2005). Fate of bacteria, Aeromonas caviae, in the midgut of the housefly Musca domestica. Inverteb. Biol. 124, 74–78. doi: 10.1111/j.1744-7410.2005.1241-09.x

[ref81] Nguyen S. G. Raza S. Ta L. T. Le L.-A. T. Ho C. T. Unno T. (2022). Metagenomic investigation of the seasonal distribution of bacterial community and antibiotic-resistant genes in Day River downstream, Ninh Binh Vietnam. Appl. Biol. Chem. 65, 1–13. doi: 10.1186/s13765-022-00687-w

[ref82] Nikolaidis I. Favini-Stabile S. Dessen A. (2014). Resistance to antibiotics targeted to the bacterial cell wall. Protein Sci. 23, 243–259. doi: 10.1002/pro.2414, PMID: 24375653 PMC3945833

[ref83] Nishino K. Senda Y. Yamaguchi A. (2008). CRP regulator modulates multidrug resistance of Escherichia coli by repressing the mdtEF multidrug efflux genes. J. Antibiot. 61, 120–127. doi: 10.1038/ja.2008.120, PMID: 18503189

[ref84] Ovchinnikov S. Kinch L. Park H. Liao Y. Pei J. Kim D. E.. (2015). Large-scale determination of previously unsolved protein structures using evolutionary information. elife 4:e09248. doi: 10.7554/eLife.09248, PMID: 26335199 PMC4602095

[ref85] Öztürk D. Adanır R. Türütoğlu H.. (2007). Isolation and antibiotic susceptibility of Aeromonas hydrophila in a carp (*Cyprinus carpio*) hatchery farm. 51.3

[ref86] Page A. J. Cummins C. A. Hunt M. Wong V. K. Reuter S. Holden M. T.. (2015). Roary: rapid large-scale prokaryote pan genome analysis. Bioinformatics 31, 3691–3693. doi: 10.1093/bioinformatics/btv421, PMID: 26198102 PMC4817141

[ref87] Palu A. P. Gomes L. M. Miguel M. A. L. Balassiano I. T. Queiroz M. L. P. Freitas-Almeida A. C.. (2006). Antimicrobial resistance in food and clinical Aeromonas isolates. Food Microbiol. 23, 504–509. doi: 10.1016/j.fm.2005.07.002 16943044

[ref88] Park S. Y. Lim S. R. Son J. S. Kim H. K. Yoon S.-W. Jeong D. G.. (2018). Complete genome sequence of Aeromonas rivipollensis KN-mc-11N1, isolated from a wild nutria (Myocastor coypus) in South Korea. Microbiol. Resour. Announce. 7, e00907–e00918. doi: 10.1128/MRA.00907-18 PMC6256423 30533878

[ref89] Parker J. L. Shaw J. G. (2011). Aeromonas spp. clinical microbiology and disease. J. Infect. 62, 109–118. doi: 10.1016/j.jinf.2010.12.003 21163298

[ref90] Patil P. K. Mishra S. S. Pradhan P. K. Manna S. K. Abraham J. T. Solanki H. G.. (2022). Usage pattern of chemicals, biologicals and veterinary medicinal products in Indian aquaculture. Rev. Aquac. 14, 2038–2063. doi: 10.1111/raq.12688

[ref91] Pérez-Duque A. Gonzalez-Muñoz A. Arboleda-Valencia J. Vivas-Aguas L. J. Córdoba-Meza T. Rodriguez-Rey G. T.. (2021). Comparative genomics of clinical and environmental isolates of vibrio spp. of Colombia: implications of traits associated with virulence and resistance. Pathogens 10:1605. doi: 10.3390/pathogens10121605, PMID: 34959560 PMC8706872

[ref92] Pérez-Valdespino A. Fernández-Rendón E. Curiel-Quesada E. (2009). Detection and characterization of class 1 integrons in Aeromonas spp. isolated from human diarrheic stool in Mexico. J. Basic Microbiol. 49, 572–578. doi: 10.1002/jobm.200900095, PMID: 19810047

[ref93] Pidiyar V. Kaznowski A. Narayan N. B. Patole M. Shouche Y. S. (2002). Aeromonas culicicola sp. nov., from the midgut of Culex quinquefasciatus. Int. J. Syst. Evol. Microbiol. 52, 1723–1728. doi: 10.1099/00207713-52-5-1723 12361279

[ref94] Piotrowska M. Dziewit L. Ostrowski R. Chmielowska C. Popowska M. (2020). Molecular characterization and comparative genomics of IncQ-3 plasmids conferring resistance to various antibiotics isolated from a wastewater treatment plant in Warsaw (Poland). Antibiotics 9:613. doi: 10.3390/antibiotics9090613, PMID: 32957637 PMC7557826

[ref95] Praveen P. Debnath C. Pramanik A. Shekhar S. Dalai N. (2014). Incidence and biochemical characterization of Aeromonas species isolated from retail fish and chicken in North Kolkata region. J. Cell Tissue Res. 14:4609.

[ref96] Ragupathi N. K. D. Sethuvel D. P. M. Anandan S. Murugan D. Asokan K. Mohan R. G. N.. (2020). First hybrid complete genome of Aeromonas veronii reveals chromosome-mediated novel structural variant mcr-3.30 from a human clinical sample. Access Microbiol. 2:acmi000103. doi: 10.1099/acmi.0.000103, PMID: 33005867 PMC7523623

[ref97] Rahimi L. E. Nene S.. (2006). The prevalence of *Aeromonas hydrophila*-induced diarrhoea in the pig, buffalo and human in Pune area. Journal of Veterinary Research, 7, 53–58.

[ref98] Rahman T. Yarnall B. Doyle D. A. (2017). Efflux drug transporters at the forefront of antimicrobial resistance. Eur. Biophys. J. 46, 647–653. doi: 10.1007/s00249-017-1238-2, PMID: 28710521 PMC5599465

[ref99] Ramadan H. Ibrahim N. Samir M. Abd El-Moaty A. Gad T. (2018). Aeromonas hydrophila from marketed mullet (Mugil cephalus) in Egypt: PCR characterization of β-lactam resistance and virulence genes. J. Appl. Microbiol. 124, 1629–1637. doi: 10.1111/jam.13734, PMID: 29453863

[ref100] Ramsamy Y. Amoako D. G. Abia A. L. K. Allam M. Ismail A. Mtshali P. S.. (2021). First genome sequence of Aeromonas hydrophilia novel sequence type 658 strain isolated from livestock in South Africa. J. Glob. Antimicrob. Resist. 24, 175–177. doi: 10.1016/j.jgar.2020.12.021, PMID: 33460845

[ref101] Ramsamy Y. Mlisana K. P. Amoako D. G. Abia A. L. K. Allam M.. (2020). Comparative pathogenomics of *Aeromonas veronii* from pigs in South Africa: dominance of the novel ST657 clone. Microorganisms 8:2008. doi: 10.3390/microorganisms8122008, PMID: 33339176 PMC7765573

[ref102] Ranjbar R. Salighehzadeh R. Sharifiyazdi H. (2019). Antimicrobial resistance and incidence of integrons in Aeromonas species isolated from diseased freshwater animals and water samples in Iran. Antibiotics 8:198. doi: 10.3390/antibiotics8040198, PMID: 31661794 PMC6963716

[ref103] Rhodes G. Parkhill J. Bird C. Ambrose K. Jones M. C. Huys G.. (2004). Complete nucleotide sequence of the conjugative tetracycline resistance plasmid pFBAOT6, a member of a group of IncU plasmids with global ubiquity. Appl. Environ. Microbiol. 70, 7497–7510. doi: 10.1128/AEM.70.12.7497-7510.2004, PMID: 15574953 PMC535204

[ref104] Richardson C. J. Robinson J. O. Wagener L. B. Burke V. (1982). *In-vitro* susceptibility of Aeromonas spp. to antimicrobial agents. J. Antimicrob. Chemother. 9, 267–274. doi: 10.1093/jac/9.4.267 7085531

[ref105] Roy A. Abraham T. J. Singha J. Julinta R. B. Boda S. (2021). Efficacy of oral oxytetracycline therapy against Aeromonas caviae infection in Nile tilapia *Oreochromis niloticus* (L.) juveniles. Journal of. Fisheries 9:93206. doi: 10.17017/j.fish.361

[ref106] Roy R. P. Bahadur M. Barat S. (2013). Isolation, identification and antibiotic resistance of Aeromonas spp. and salmonella spp. from the fresh water loach, Lepidocephalichthys guntea and water of Terai River Lotchka, West Bengal, India. Zoo. Poloniae 58, 5–17. doi: 10.2478/zoop-2013-0001

[ref107] Roy R. P. Barat S. (2011). Influence of water quality on the bacterial contamination of resident loach, Lepidocephalichthys guntea (Hamilton Buchanan) and on a Terai River Lotchka of Darjeeling District, West Bengal, India. Environ. Sci. 5, 116–123.

[ref108] Saengsitthisak B. Chaisri W. Punyapornwithaya V. Mektrirat R. Klayraung S. Bernard J. K.. (2020). Occurrence and antimicrobial susceptibility profiles of multidrug-resistant aeromonads isolated from freshwater ornamental fish in Chiang Mai province. Pathogens 9:973. doi: 10.3390/pathogens9110973, PMID: 33266430 PMC7700646

[ref109] Saffari N. Salmanzadeh-Ahrabi S. Abdi-Ali A. Rezaei-Hemami M. (2016). A comparison of antibiotic disks from different sources on Quicolor and Mueller-Hinton agar media in evaluation of antibacterial susceptibility testing. Iran. J. Microbiol. 8, 307–311. 28149489 PMC5277598

[ref110] Saharia P. K. Hussain I. A. Pokhrel H. Kalita B. Borah G. Yasmin R. (2021). Prevalence of motile Aeromonas Septicaemia (MAS) in fish culture systems of the Central Brahmaputra Valley zone of Assam. India. Aquacul. Res. 52, 1201–1214. doi: 10.1111/are.14979

[ref111] Sakulworakan R. Chokmangmeepisarn P. Dinh-Hung N. Sivaramasamy E. Hirono I. Chuanchuen R.. (2021). Insight into whole genome of Aeromonas veronii isolated from freshwater fish by resistome analysis reveal extensively antibiotic resistant traits. Front. Microbiol. 12:733668. doi: 10.3389/fmicb.2021.733668, PMID: 34603262 PMC8484913

[ref112] Schar D. Klein E. Y. Laxminarayan R. Gilbert M. Van Boeckel T. P. (2020). Global trends in antimicrobial use in aquaculture. Sci. Rep. 10, 1–9. doi: 10.1038/s41598-020-78849-3 33318576 PMC7736322

[ref113] Schmidt A. S. Bruun M. S. Dalsgaard I. Larsen J. L. (2001). Incidence, distribution, and spread of tetracycline resistance determinants and integron-associated antibiotic resistance genes among motile aeromonads from a fish farming environment. Appl. Environ. Microbiol. 67, 5675–5682. doi: 10.1128/AEM.67.12.5675-5682.2001, PMID: 11722922 PMC93359

[ref114] Seemann T. (2014). Prokka: rapid prokaryotic genome annotation. Bioinformatics 30, 2068–2069. doi: 10.1093/bioinformatics/btu153, PMID: 24642063

[ref115] Seemann T.. (2016). ABRicate: Mass Screening of Contigs for Antibiotic RESISTANCE Genes. San Francisco: GitHub.

[ref116] Seetha K. Jose B. Jasthi A. Rao P. (2004). Meningitis due to *Aeromonas hydrophila*. Indian J. Med. Microbiol. 22, 191–192. doi: 10.1016/S0255-0857(21)02836-X, PMID: 17642732

[ref117] Seo K. W. Lee Y. J. (2018). Prevalence and characterization of β-lactamases genes and class 1 integrons in multidrug-resistant Escherichia coli isolates from chicken meat in Korea. Microb. Drug Resist. 24, 1599–1606. doi: 10.1089/mdr.2018.0019, PMID: 29927695

[ref118] Shannon K. King A. Phillips I. (1986). β-Lactamases with high activity against imipenem and Sch 34343 from Aeromonas hydrophila. J. Antimicrob. Chemother. 17, 45–50. doi: 10.1093/jac/17.1.45, PMID: 3485091

[ref119] Shen Y. Xu C. Sun Q. Schwarz S. Ou Y. Yang L.. (2018). Prevalence and genetic analysis of mcr-3-positive Aeromonas species from humans, retail meat, and environmental water samples. Antimicrob. Agents Chemother. 62, e00404–e00418. doi: 10.1128/AAC.00404-18 29967026 PMC6125509

[ref120] Singh V. Rathore G. Kapoor D. Mishra B. Lakra W. (2008). Detection of aerolysin gene in Aeromonas hydrophila isolated from fish and pond water. Indian J. Microbiol. 48, 453–458. doi: 10.1007/s12088-008-0056-8, PMID: 23100746 PMC3476786

[ref121] Singhal N. Kumar M. Kanaujia P. K. Virdi J. S. (2015). MALDI-TOF mass spectrometry: an emerging technology for microbial identification and diagnosis. Front. Microbiol. 6:791. doi: 10.3389/fmicb.2015.00791 26300860 PMC4525378

[ref122] Sinha S. Shimada T. Ramamurthy T. Bhattacharya S. Yamasaki S. Takeda Y. (2004). Prevalence, serotype distribution, antibiotic susceptibility and genetic profiles of mesophilic Aeromonas species isolated from hospitalized diarrhoeal cases in Kolkata. India. J. Med. Microbiol. 53, 527–534. doi: 10.1099/jmm.0.05269-0, PMID: 15150333

[ref123] Sivaraman G. K. Rajan V. Vijayan A. Elangovan R. Prendiville A. Bachmann T. T. (2021). Antibiotic resistance profiles and molecular characteristics of extended-spectrum beta-lactamase (ESBL)-producing *Escherichia coli* and Klebsiella pneumoniae isolated from shrimp aquaculture farms in Kerala, India. Front. Microbiol. 12:622891. doi: 10.3389/fmicb.2021.622891, PMID: 34489875 PMC8417373

[ref124] Smith T. Walker E. Kaufman M. (1998). Bacterial density and survey of cultivable heterotrophs in the surface water of a freshwater marsh habitat of Anopheles quadrimaculatus larvae (Diptera: Culicidae). J. Am. Mosq. Control Assoc. 14, 72–77. 9599327

[ref125] Sørum H. L’Abée-Lund T. M. Solberg A. Wold A. (2003). Integron-containing IncU R plasmids pRAS1 and pAr-32 from the fish pathogen Aeromonas salmonicida. Antimicrob. Agents Chemother. 47, 1285–1290. doi: 10.1128/AAC.47.4.1285-1290.2003, PMID: 12654659 PMC152485

[ref126] Stalder T. Barraud O. Casellas M. Dagot C. Ploy M.-C. (2012). Integron involvement in environmental spread of antibiotic resistance. Front. Microbiol. 3:119. doi: 10.3389/fmicb.2012.00119 22509175 PMC3321497

[ref127] Stanhope M. J. Lefébure T. Walsh S. L. Becker J. A. Lang P. Bitar P. D. P.. (2008). Positive selection in penicillin-binding proteins 1a, 2b, and 2x from Streptococcus pneumoniae and its correlation with amoxicillin resistance development. Infect. Genet. Evol. 8, 331–339. doi: 10.1016/j.meegid.2008.02.001, PMID: 18394970

[ref128] Studer N. Frey J. Vanden Bergh P. (2013). Clustering subspecies of *Aeromonas salmonicida* using IS630typing. BMC Microbiol. 13, 1–12. doi: 10.1186/1471-2180-13-36 23406017 PMC3608246

[ref129] Subashkumar R. Thayumanavan T. Vivekanandhan G. Lakshmanaperumalsamy P. (2006). Occurrence of Aeromonas hydrophila in acute gasteroenteritis among children. Indian J. Med. Res. 123, 61–66. 16567870

[ref130] Sudheer Khan S. Bharath Kumar E. Mukherjee A. Chandrasekaran N. (2011). Bacterial tolerance to silver nanoparticles (SNPs): *Aeromonas punctata* isolated from sewage environment. J. Basic Microbiol. 51, 183–190. doi: 10.1002/jobm.201000067, PMID: 21077112

[ref131] Tang L. Huang J. She J. Zhao K. Zhou Y. (2020). Co-occurrence of the blaKPC-2 and Mcr-3.3 gene in Aeromonas caviae SCAc2001 isolated from patients with diarrheal disease. Infect. Drug Resist. 13, 1527–1536. doi: 10.2147/IDR.S245553, PMID: 32547122 PMC7259443

[ref132] Tarumoto N. Sakai J. Sujino K. Yamaguchi T. Ohta M. Yamagishi J.. (2017). Use of the Oxford Nanopore MinION sequencer for MLST genotyping of vancomycin-resistant enterococci. J. Hosp. Infect. 96, 296–298. doi: 10.1016/j.jhin.2017.02.020, PMID: 28389092

[ref133] Tatusova T. DiCuccio M. Badretdin A. Chetvernin V. Nawrocki E. P. Zaslavsky L.. (2016). NCBI prokaryotic genome annotation pipeline. Nucleic Acids Res. 44, 6614–6624. doi: 10.1093/nar/gkw569, PMID: 27342282 PMC5001611

[ref134] Tóth A. G. Csabai I. Maróti G. Jerzsele Á. Dubecz A. Patai Á. V.. (2020). A glimpse of antimicrobial resistance gene diversity in kefir and yoghurt. Sci. Rep. 10, 1–12. doi: 10.1038/s41598-020-80444-5 33384459 PMC7775456

[ref135] Tran T. T. Scott A. Tien Y.-C. Murray R. Boerlin P. Pearl D. L.. (2021). On-farm anaerobic digestion of dairy manure reduces the abundance of antibiotic resistance-associated gene targets, and the potential for plasmid transfer. Appl. Environ. Microbiol. 87, 02980–02920. doi: 10.1128/AEM.02980-20 PMC8231723 33931422

[ref136] Turutoglu H. Ercelik S. Corlu M. (2005). Aeromonas hydrophila-associated skin lesions and septicaemia in a Nile crocodile (*Crocodylus niloticus*): clinical communication. J. S. Afr. Vet. Assoc. 76, 40–42. doi: 10.4102/jsava.v76i1.393, PMID: 15900900

[ref137] Tyagi A. Sharma C. Srivastava A. Kumar B. N. Pathak D. Rai S. (2022). Isolation, characterization and complete genome sequencing of fish pathogenic Aeromonas veronii from diseased *Labeo rohita*. Aquaculture 738085. doi: 10.1016/j.aquaculture.2022.738085

[ref138] Ullah S. R. Majid M. Andleeb S. (2020). Draft genome sequence of an extensively drug-resistant neonatal Klebsiella pneumoniae isolate harbouring multiple plasmids contributing to antibiotic resistance. J. Glob. Antimicrob. Resist. 23, 100–101. doi: 10.1016/j.jgar.2020.08.008, PMID: 32866642

[ref139] Van Boeckel T. P. Pires J. Silvester R. Zhao C. Song J. Criscuolo N. G.. (2019). Global trends in antimicrobial resistance in animals in low-and middle-income countries. Science 365:eaaw1944. doi: 10.1126/science.aaw1944, PMID: 31604207

[ref140] Vaseeharan B. Ramasamy P. Murugan T. Chen J. (2005). *In vitro* susceptibility of antibiotics against vibrio spp. and Aeromonas spp. isolated from Penaeus monodon hatcheries and ponds. Int. J. Antimicrob. Agents 26, 285–291. doi: 10.1016/j.ijantimicag.2005.07.005, PMID: 16139992

[ref141] Vásquez-Ponce F. Higuera-Llantén S. Parás-Silva J. Gamboa-Acuña N. Cortés J. Opazo-Capurro A.. (2022). Genetic characterization of clinically relevant class 1 integrons carried by multidrug resistant bacteria (MDRB) isolated from the gut microbiota of highly antibiotic treated Salmo salar. J. Glob. Antimicrob. Resist. 29, 55–62. doi: 10.1016/j.jgar.2022.02.003, PMID: 35158077

[ref142] Vincent A. T. Rouleau F. D. Moineau S. Charette S. J. (2017). Study of mesophilic Aeromonas salmonicida A527 strain sheds light on the species’ lifestyles and taxonomic dilemma. FEMS Microbiol. Lett. 364:fnx239. doi: 10.1093/femsle/fnx239 29126137

[ref143] Vincent A. T. Trudel M. V. Freschi L. Nagar V. Gagné-Thivierge C. Levesque R. C.. (2016). Increasing genomic diversity and evidence of constrained lifestyle evolution due to insertion sequences in Aeromonas salmonicida. BMC Genom. 17, 1–12. doi: 10.1186/s12864-016-2381-3 PMC4709979 26753691

[ref144] Vivekanandhan G. Hatha A. Lakshmanaperumalsamy P. (2005). Prevalence of *Aeromonas hydrophila* in fish and prawns from the seafood market of Coimbatore. South India. Food Microbiol. 22, 133–137. doi: 10.1016/j.fm.2004.01.015

[ref145] Von Graevenitz A. Mensch A. H. (1968). The genus Aeromonas in human bacteriology: report of 30 cases and review of the literature. N. Engl. J. Med. 278, 245–249. doi: 10.1056/NEJM196802012780504 5635458

[ref146] Walia K. Sharma M. Vijay S. Shome B. R. (2019). Understanding policy dilemmas around antibiotic use in food animals & offering potential solutions. Indian J. Med. Res. 149, 107–118. doi: 10.4103/ijmr.IJMR_2_18, PMID: 31219075 PMC6563746

[ref147] Walsh T. R. Stunt R. A. Nabi J. A. MacGowan A. Bennett P. (1997). Distribution and expression of beta-lactamase genes among Aeromonas spp. J. Antimicrob. Chemother. 40, 171–178. doi: 10.1093/jac/40.2.171, PMID: 9301981

[ref148] Wamala S. P. Mugimba K. K. Dubey S. Takele A. Munang’andu H. M. Evensen Ø.. (2018). Multilocus sequence analysis revealed a high genotypic diversity of *Aeromonas hydrophila* infecting fish in Uganda. J. Fish Dis. 41, 1589–1600. doi: 10.1111/jfd.12873, PMID: 30074242

[ref149] Wang L. Fu L. Liu Z. Guo H. Wang L. Feng M.. (2019). Comparative analysis of antimicrobial resistance, integrons, and virulence genes among extended-spectrum β-lactamase-positive Laribacter hongkongensis from edible frogs and freshwater fish. Microb. Drug Resist. 25, 855–864. doi: 10.1089/mdr.2018.0366, PMID: 30767721

[ref150] Wang Y. Hou N. Rasooly R. Gu Y. He X. (2021). Prevalence and genetic analysis of chromosomal mcr-3/7 in Aeromonas from US animal-derived samples. Front. Microbiol. 12:1029. doi: 10.3389/fmicb.2021.667406 PMC8120114 33995332

[ref151] Wang B. Mao C. Feng J. Li Y. Hu J. Jiang B.. (2021). A first report of Aeromonas veronii infection of the sea bass, Lateolabrax maculatus in China. Fronti. Vet. Sci. 7:600587. doi: 10.3389/fvets.2020.600587, PMID: 33553279 PMC7855973

[ref152] Xiao X. Jiang Y. Yang X. Zheng J. Guo Z. Qi Q.. (2020). Nosocomial outbreak of Aeromonas hydrophila surgical site infections after spinal surgery: Identification and control. doi: 10.21203/rs.2.20684/v1

[ref153] Yu K. Wang H. Cao Z. Gai Y. Liu M. Li G.. (2022). Antimicrobial resistance analysis and whole-genome sequencing of salmonella enterica serovar Indiana isolate from ducks. J. Glob. Antimicrob. Resist. 28, 78–83. doi: 10.1016/j.jgar.2021.12.013, PMID: 34942402

[ref154] Zago V. Veschetti L. Patuzzo C. Malerba G. Lleo M. M. (2020). Resistome, mobilome and virulome analysis of Shewanella algae and vibrio spp. strains isolated in Italian aquaculture centers. Microorganisms 8:572. doi: 10.3390/microorganisms8040572, PMID: 32326629 PMC7232470

[ref155] Zdanowicz M. Mudryk Z. J. Perliński P. (2020). Abundance and antibiotic resistance of Aeromonas isolated from the water of three carp ponds. Vet. Res. Commun. 44, 9–18. doi: 10.1007/s11259-020-09768-x, PMID: 31965460 PMC7040064

[ref156] Zemelman R. Gonzalez C. Mondaca M. A. Silva J. Merino C. Dominguez M. (1984). Resistance of Aeromonas hydrophila to β-lactam antibiotics. J. Antimicrob. Chemother. 14, 575–579. doi: 10.1093/jac/14.6.575, PMID: 6335147

[ref157] Zhong Y. Guo S. Schlundt J. (2021). Reservoir water in Singapore contains ESBL-producing and carbapenem-resistant bacteria with conjugatable conserved gene cluster transfer between different species. bioRxiv. doi: 10.1101/2021.06.13.448270

