# Supplementary material for: Aeromonas species isolated from aquatic organisms, insects, chicken, and humans in India show similar antimicrobial resistance profiles
Source: Front Microbiol. 2022 Dec 1;13:1008870. doi: 10.3389/fmicb.2022.1008870 (PMC9752027; doi:10.3389/fmicb.2022.1008870)
Supplement: Supplementary file 1 [file Table_1.DOCX]

Table S1. Plasmid genes detected in the *Aeromonas* genomes


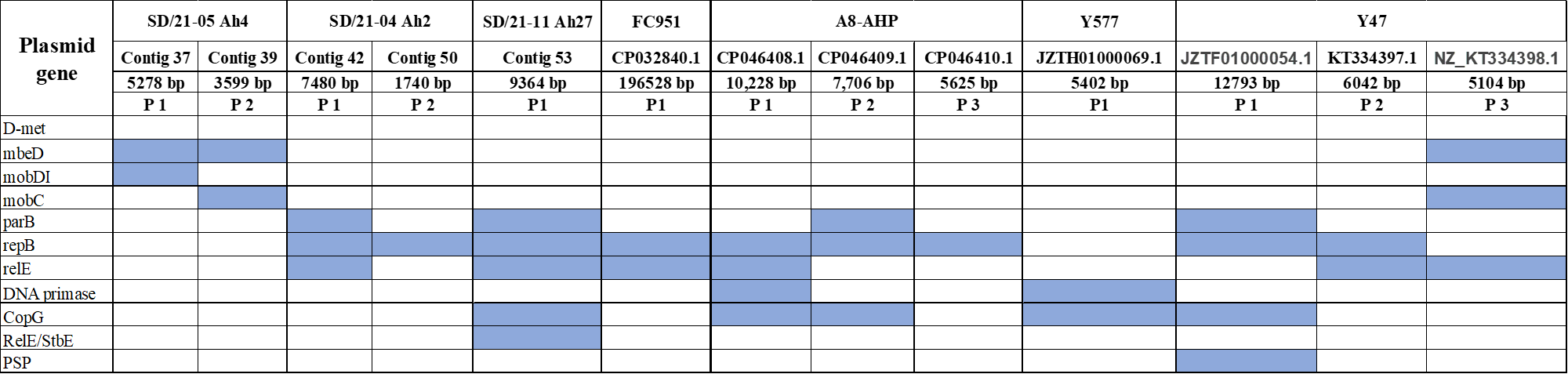


All plasmids were identified using Plasmidfinder v 2.0 (Ullah et al., 2020) in the Aeromonas genomes

Blue = presence of genes (detected) White/blank=absence of gene (not detected)
